# Semaphorin3f as a cardiomyocyte derived regulator of heart chamber development

**DOI:** 10.1186/s12964-022-00874-8

**Published:** 2022-08-19

**Authors:** Rami Halabi, Paula Bernice Cechmanek, Carrie Lynn Hehr, Sarah McFarlane

**Affiliations:** Hotchkiss Brain Institute, Alberta Children’s Hospital Research Institute, Department of Cell Biology and Anatomy, University of Calgary, 3330 Hospital Dr., NW, Calgary, AB T2N 4N1 Canada

**Keywords:** Zebrafish, Ventricle, Atrium, Semaphorin, Plexin, Embryo, Edema, CRISPR mutant

## Abstract

**Background:**

During development a pool of precursors form a heart with atrial and ventricular chambers that exhibit distinct transcriptional and electrophysiological properties. Normal development of these chambers is essential for full term survival of the fetus, and deviations result in congenital heart defects. The large number of genes that may cause congenital heart defects when mutated, and the genetic variability and penetrance of the ensuing phenotypes, reveals a need to understand the molecular mechanisms that allow for the formation of chamber-specific cardiomyocyte differentiation.

**Methods:**

We used in situ hybridization, immunohistochemistry and functional analyses to identify the consequences of the loss of the secreted semaphorin, Sema3fb, in the development of the zebrafish heart by using two *sema3fb* CRISPR mutant alleles.

**Results:**

We find that in the developing zebrafish heart *sema3fb* mRNA is expressed by all cardiomyocytes, whereas mRNA for a known receptor Plexina3 (Plxna3) is expressed preferentially by ventricular cardiomyocytes. In *sema3fb* CRISPR zebrafish mutants, heart chamber development is impaired; the atria and ventricles of mutants are smaller in size than their wild type siblings, apparently because of differences in cell size and not cell numbers. Analysis of chamber differentiation indicates defects in chamber specific gene expression at the border between the ventricular and atrial chambers, with spillage of ventricular chamber genes into the atrium, and vice versa, and a failure to restrict specialized cardiomyocyte markers to the atrioventricular canal (AVC). The hypoplastic heart chambers are associated with decreased cardiac output and heart edema.

**Conclusions:**

Based on our data we propose a model whereby cardiomyocytes secrete a Sema cue that, because of spatially restricted expression of the receptor, signals in a ventricular chamber-specific manner to establish a distinct border between atrial and ventricular chambers that is important to produce a fully functional heart.

**Video abstract**

**Supplementary information:**

The online version contains supplementary material available at 10.1186/s12964-022-00874-8.

## Background

The heart is the first organ to form and become functional in an embryo, and congenital heart defects affect 9 per 1000 live births [1]. Collectively, congenital heart defects involve the irregular formation of heart chambers and/or valves. In vertebrates, chamber morphogenesis follows the twisting, expansion and septation of the linear heart tube into a two- (teleost), three- (amphibian) or four-(mammalian/avian) chambered heart [2]. These cardiac chambers, the atrium and ventricle, differ in their biochemical properties, electrophysiological and contractile capacities, as well as in their transcriptional profiles [3, 4]. What maintains the segregation of the cell populations in the two discrete chambers as the embryonic heart differentiates and undergoes morphogenesis is unknown.

During development, cells within tissues acquire unique identities, and need to be segregated from one another within subdivisions of the tissue [5, 6]. This is true for example of the developing hindbrain, where the initial expression of genes within adjacent rhombomeres is initially imprecise, and is sharpened as embryogenesis proceeds, producing domains with straight borders [6]. Key in this process are interactions between neighbouring hindbrain cells that involve contact-mediated repellent signaling via Ephrin ligands and Eph receptors. Whether a similar process of refinement of the border between ventricular and atrial chambers of the heart occurs is unknown. Chamber-specific development depends on extrinsic factors, which include Nodal, Notch, Bone morphogenetic protein (Bmp), Fibroblast growth factor (Fgf) and retinoic acid [7–9]. These signals then pattern the early heart tube to establish secondary localized signaling that spatially restricts the expression of differentiation genes to the specific chambers and valves. Intrinsic regulators, such as transcription factors, are also necessary for chamber-specific cardiomyocyte development. For example, ventricle specific expression of Irx4 is necessary in chick for the expression of ventricular Myosin Heavy Chain I and suppression of atrial Myosin Heavy Chain [10]. Further, PITX2 within the murine left atrium inhibits the expression of *Shox2* and the specification of the pacemaker cells of the sinoatrial node [11]. An unanswered question is whether active mechanisms are required to maintain segregation of the two populations of cardiomyocytes once established, especially considering the disruptive forces of cardiac chamber morphogenesis and heart looping.

One group of molecules known to act as repellents for developing cells of the nervous and cardiovascular systems are the secreted class 3 Semaphorins (Sema3s), which are used as guidance cues for vessels, axons and neural crest cells [12–15]. Sema3 signaling is mediated through the canonical Plexin (Plxn) receptors and their coreceptors, the Neuropilins (Nrp) [16]. Sema3s are implicated in murine cardiovascular development via their regulation of the directed movement of associated cells [17]. Sema3s pattern embryonic vessels by directing migrating endothelial cells [18], and play various roles in heart development: Sema3a regulates angioblast migration to form the dorsal aorta in zebrafish [19] and sympathetic innervation of the mouse heart [20], murine neural crest cells and zebrafish epicardial cells require Sema3c and Sema3f, respectively, to move into the heart outflow tract [21, 22], Sema3d promotes migration of neural crest cells into the primary heart field in zebrafish [23], and Sema3e-mediated PlxnD1 signaling in endothelial cells is required for ventricular compaction in the mouse heart [24]. Whether Sema3s play roles in heart formation via the direct regulation of cardiomyocyte development is unknown.

Here, we take advantage of the zebrafish model as a powerful tool to study the development of the heart; the embryos do not require a functional cardiovascular system during embryogenesis [25], which allows for the extended characterization of manipulations. We characterized *sema3* expression in zebrafish embryos and found mRNA for secreted Sema3fb in cardiomyocytes during chamber differentiation. This expression was of particular significance given that we find *nrp2b* and *plxna3* receptor mRNAs are expressed preferentially by ventricular cardiomyocytes, suggesting spatially restricted Sema3fb signaling within the developing myocardium. To investigate a role for ventricular cardiomyocyte Sema3fb signaling, we generated CRISPR/Cas9 zebrafish *sema3fb* mutants. With loss of either Sema3fb, or signaling through its Plxna3 receptor via morpholino-oligonucleotide knockdown, chamber specific gene expression is either lost from or expands beyond the border between the atrium and ventricle. Accompanying the loss of segregated gene expression between the cardiac chambers is a decrease in the size of both chambers, and significantly impaired cardiac output and edema of the early post-embryonic heart. We propose a model whereby Sema3fb signaling ensures segregation of atrial and ventricular cardiomyocyte chambers, a feature that appears critical for chamber development and heart function.

## Methods

### Zebrafish strains and maintenance

Zebrafish (Danio rerio) were maintained on a 14-h light/10-h dark cycle at 28 °C. Embryos were obtained by natural spawning, raised in E3 medium supplemented with 0.25 mg/L methylene blue, and staged by convention [26, 27]. To permit exact developmental staging, adult zebrafish spawned for 10 min only before embryo collection. Dishes were screened at 6 h post fertilization (hpf) to remove unfertilized eggs or embryos that showed delayed embryogenesis. We used *sema3fb*^*ca305* (28)^ and *sema3fb*^*ca306*^ genetic mutants. These lines were maintained as heterozygotes for all initial experiments, and thereafter maintained as homozygotes and wild type (WT) siblings for future embryo collection. The *Tg(-6.5kdrl:mCherry)*^*ci5*^ line was used as an outcross for *sema3fb*^*ca305*^ mutants to label endothelial cells [29], *Tg(flk:EGFP)* to label endothelial cells, and *Tg(myl7:EGFP)* [30] Casper fish for identification of cardiomyocytes. Sex was not a variable in these studies as zebrafish cannot be sexed as embryos.

### sema3fb mutant lines

To generate genetic knock-outs by using the CRISPR/Cas9 system a sgRNA (5’—GAAGGACAAGAAGACCCGCG) targeting *sema3fb* exon 2 was selected following CHOPCHOP query [31] and analysis of secondary structure using Vienna RNAfold Prediction (rna.tbi.univie.ac.at). sgRNA template generation and transcription was carried out in accordance to the protocol described previously [32]. One-cell stage embryos were injected with a 1 nl mix of 56–60 pg sgRNA and 190 pg *cas9* mRNA (Addgene plasmid #47,322). Mosaic embryos were raised to adulthood and crossed with Tupel Long fin fish to identify founders. To genotype, caudal fin clippings were collected from tricaine methanesulfonate (MS-222; 160 mg/L) anesthetized adults. Genomic DNA extraction was performed [33] and amplified by PCR using primers (Forward: 5’- ATTGCCCCACAAAATAACATTC; Reverse: 5’—GTCTACTCTGTGAATTTCCCGC) around the expected mutation site. Amplicons were sequenced at University of Calgary DNA Core facility for definitive genotype confirmation.

### Quantitative real time PCR

Total RNA from embryos was prepared using the TRIzol reagent (Invitrogen) extraction method [34]. Embryonic hearts at specific developmental ages were collected in Trizol and homogenized using a 26½ gauge syringe. First strand cDNA was made using the Superscript II RT-PCR (Invitrogen, 11,904–018) protocol with 100 ng of total RNA for conversion. Each RT-qPCR reaction had a 10 μl final volume containing 0.5 μl of cDNA, 500 nM of each primer and 5 μl of SYBR Green QuantiFast RT-qPCR master mix (Qiagen). Primers used were: *bmp4a* (F: 5’ – GACCCGTTTTACCGTCTTCA; R: 5’- TTTGTCGAGAGGTGATGCAG); *irx1a* (F: 5’- CAAGATGACCCTCACGCAAG; R: 5’- CCAGGTCACCTTGTTCTCCT); *tbx5a* (F: 5’- AACCATCTGGATCCCTTCG; R: 5’- TGTTTTCATCCGCCTTGAC). An Applied Biosystems QuantiStudio 6 Real-Time PCR system was used, and gene expression was normalized relative to *ß-actin* (F: 5’-GCAGAAGGAGATCACATCCCTGGC; R: 5’- CATTGCCGTCACCTTCACCGTTC). Reactions were performed in three technical replicates, and all results made from 3–4 independent biological replicates.

### in situ  hybridization

Digoxigenin RNA probes were synthesized from plasmid templates or via PCR products containing an RNA polymerase binding sequence on the reverse primer [35]. Probes used included: *bmp4a* (5’- CTGCCAGGACCACGTAACAT; 5’-GAAATTAATACGACTCACTATAGGGTGGCGCCTTTAACACCTCAT); *fgf8a* (5’-ATTTCAGTCGTCCGCTTTC; 5’-GGATCCATTAACCCTCACTAAAGGGAAAACATTGCTTTGCTGTGATG); *irx1a* (5’- GAGAACAAGGTGACCTGGGG; 5’-GAAATTAATACGACTCACTATAGGGTGAAGAGGACGAAACGACGA); *myh6* (5’- GGAGTACGTGAAGGGGCAAA; 5’-GAAATTAATACGACTCACTATAGGGGCTCGTCCCGAAATGAATGC); *myh7* (5’- GCAACTTGGTGAGGGAGGAA; 5’-GAAATTAATACGACTCACTATAGGGAGCAAGCTTACGGCCTCTTT); *nfatc1* (5’-TCAATTCCTCTGGGCAACCC; 5’GAAATTAATACGACTCACTATAGGGAGAATTTGGACGGGAACCCC); *plxna1b* (5’-GACAAGCTGAGAAACCCTCCCCG; 5’-TTTAAATCAAAGCACATTTTATCTAAAGAAAATTG); *tbx2b* (5’-GTACAGTGGACAGGGCAAGA; 5’-TAATACGACTCACTATAGCGTGACTCAAAGCCGGATGGA); *hand* 2 (a kind gift from Dr. Sarah Childs); *notch1b* (a kind gift from Dr. Peng Huang, (pCRII, linearized with EcoRV)); *plxna3* (pCRII, linearized with EcoRV); *sema3fb* (pCR4, linearized with NotI); *tbx5a* (pCRII, linearized with EcoRV); *tnnt2a* (pCRII, linearized with EcoRV); *sema3fa* and *npnrp2b* as described previously [28]. Whole mount in situ hybridization (ISH) was performed as described previously with some minor modifications [35]. The 2xSSC step was carried out at 70 °C and 0.2 × SSC at room temperature. Embryos were incubated directly in anti-DIG-AP, Fab fragment antibody (Roche) solution in blocking buffer at room temperature for 2 h with no prior blocking step. NBT/BCIP (Roche) was used at a 4.5/3.5 μl/ml ratio to stain embryos. Stained embryos were fixed in 4% paraformaldehyde (PFA) and washed in PBS with 0.1% Triton X-100 (PBST). To directly compare ISH signals between genotypes, all embryos were processed in the same tube.

### Immunolabeling and imaging

Embryos were fixed overnight in 4% PFA/PBS. Samples were blocked in PBST with 10% bovine serum albumin and 2% normal sheep serum for 30 min and transferred to primary antibody solution in 1/10 blocking buffer overnight at 4 °C. Samples were washed with PBST and incubated in secondary antibody (Alexa Fluor 488/555 rabbit/mouse) supplemented with 5 mg/ml Hoechst in PBST for 45 min. Embryos were fixed and imaged by confocal microscopy. The following primary antibodies were used: MF20 (1:500, Developmental Studies Hybridoma Bank (DSHB)), pHH3 (1:500, Millipore), S46 (1:10, DSHB), zn8 (DM-GRASP; 1:500, DSHB). ApopTag Peroxidase In Situ Apoptosis Detection kit (Millipore) was used to detect apoptotic cells in embryos, with overnight diaminobenzidine staining. For confocal microscopy, embryos were imaged with a 10 × or 20 × objective on a Zeiss LSM 700 microscope after mounting in 0.8–1% low melting point agarose (Invitrogen) on a glass bottom dish. Optical slices were taken at intervals from 1–5 μm and subjected to 2 times averaging. Z-stacks were processed in Zen Blue as maximal projections and compiled using Adobe Photoshop 2020. For recovery of the *myl7:EGFP* or *flk:eGFP* transgene following ISH, embryos were put into acetone at -20 °C for 20 min followed by 3 × PBST washes. Embryos were blocked for 1 h with 10% normal sheep serum in PBST then incubated overnight at 4 °C with JL-8 (1:500, Clontech Laboratories Inc). Secondary antibody was added as above before embedding the samples in JB4 medium (Polysciences) and sectioning at 7 µm with a Leica microtome (Leica).

### Quantitation of in situ hybridization expression domains

WT and mutant hearts were imaged at 48 hpf from a ventral view of the embryo. Images were blinded as to genotype, and brightness and contrast adjusted on individual images to maximize the resolution of the domain above background. A line drawn across the morphological constriction between the atrium and ventricle was used as the atrioventricular border. Either FIJI [36] or Axiovision (Carl Zeiss Microscopy) software were used to encircle the expression domain and measure the domain area. Schematics (Fig. 4, Additional file 1: Fig. 4) indicate how *myh7* positive ventricular length and width were measured.

### Heart function and morphology analysis

Embryos (72 hpf) were anaesthetized with minimal MS-222 and positioned on their right side laterally on a glass slide in a water droplet. Video images were taken 30 frames/second for 15 secs using a Leica DM5500 B microscope equipped with a Leica DFC365 FX. Heart rate was determined in a 15 s window. Measurements [37] were made from frame stills during systole and diastole across three heart beats using ImageJ. Briefly, the following equations were used [2]: Ventricle volume = (0.523) (Ventricle Width) (Ventricle Length); Fractional Shortening = (100) (Ventricle Width Diastole-Ventricle Width Systole)/ Ventricle Width Diastole); Stroke Volume = Ventricle Volume Diastole – Ventricle Volume Systole. Ejection Fraction = (100) (Stroke Volume/Ventricle Volume Diastole). Cardiac Output = (Heart Rate) (Stroke Volume).

### Hematoxylin and eosin (H&E) staining

H&E staining of JB-4 plastic sections was carried out as described previously [38] with the following modifications: Acid and base washes were omitted, Harris Modified Hematoxylin Solution (Sigma) and Eosin Y (EMD) were used, and tap water replaced prescribed substitute. Slides were air dried for 15 mins [39] and coverslipped using Permount (Fischer Scientific). Myocardial ventricle wall thickness for each embryo was the average of measurements made at two points along the anterior–posterior axis from 1–2 sections stained by H&E.

### Cardiomyocyte measurements

Morphometries (area and perimeter) of confocal imaged, Zn8-immunolabelled cardiomyocytes were measured in ImageJ at 48 hpf. Cells measured had clearly visible outlines. Of note, only cells central to the outer curvature of the ventral ventricle were assessed. Circularity was used to distinguish cellular morphologies in which 1 represents a perfect circle. This was calculated by the following formula: Circularity = 4 πArea/Perimeter [2]. Measurements were averaged from 8–12 cells per embryo. Additionally, projected confocal MF-20-immunolabeled images were analyzed using ‘RGB Measure’ tool on ImageJ across the entirety of the heart, values averaged for all samples, and represented as an intensity plot across distance.

### Sema3f Overexpression and Morpholino-mediated Knockdown

For heart specific overexpression, *sema3fa* was amplified from Image Clone 9,038,816 using primers that incorporate *attb1/b2* recombination sites (5’-GGGGACAAGTTTGTACAAAAAAGCAGGCTTCACCATGCAGGGAGCCGGGACTTTGGTG-3’ and 5’-GGGGACCACTTTGTACAAGAAAGCTGGGTTTGTCTCAGCCATGCTGCTCTGCTC-3’) and inserted into pDONR221 to create a *pME-sema3fa* vector. Three-way Tol2 gateway cloning [40] was used to insert *sema3fa* downstream of the cardiomyocyte *myl7* promoter [30] giving *myl7:sema3fa:p3E-MTpA* in Tol2 with *pDest:mKate* [41] as the transgenesis marker. One cell-stage zebrafish embryos were injected with a solution consisting of 12.5–25 ng/µl plasmid with or without 25 ng/µl *transposase* mRNA, and embryos checked at 48 hpf for mKate eye expression. For knockdown of *sema3fb* we injected 1-3 ng of an antisense morpholino oligonucleotide [42] that targets the ATG start site of *sema3fb* (CATAGACTGTCCAAGAGCATGGTGC, Gene Tools LLC, Corvalis, OR, USA) into 1-cell zebrafish embryos. For knockdown of *plxna3* we injected 2-4 ng of an antisense morpholino oligonucleotide that targets the first initiation codon of *plxna3* [42]. For morpholino injections, control embryos were injected with water. 48 hpf embryos were scored for edema and fixed for ISH and/or anti-myc immunohistochemistry.

### Statistics

Data sets for qualitative scoring or quantitation were analyzed in a blinded fashion. Sample size calculations were not performed because we characterized mutants, and thus had no prediction as to the likely size of effects to use in power calculations. Results are expressed as mean ± standard error of the mean (SEM) unless otherwise indicated. Statistical analyses were performed by using Prism 8 software (Graph Pad). An unpaired, non-parametric Mann Whitney U test was used for comparisons of two samples, and for comparisons between multiple samples a One-Way ANOVA was used for normally distributed data and a Kruskal–Wallis test used if data failed to pass a normality test.

## Results

### sema3fb is expressed by developing cardiomyocytes

**Fig. 1:**
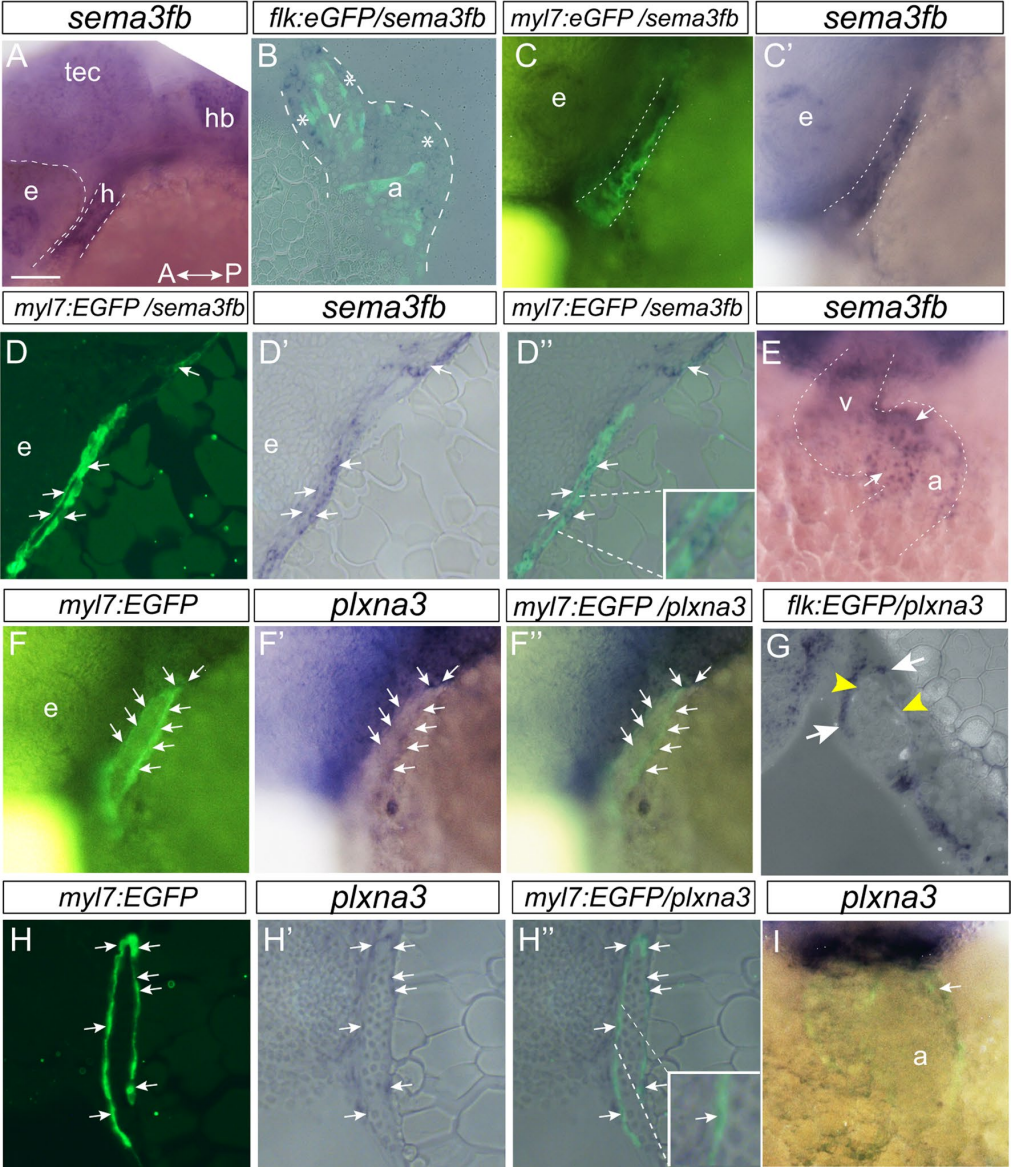
*sema3fb* is expressed by embryonic cardiomyocytes. **A-E** Whole mount (A,C,C’,E) and horizontal (B) and sagittal (D-D’’) sections of *sema3fb* ISH on 28 hpf (A,C,D), 36 hpf (B) and 48 hpf (E) zebrafish hearts. *sema3fb* is expressed through the heart chamber, in GFP positive cardiomyocytes in a *Tg(myl7:EGFP)* heart (C–C’,D-D’’) and surrounding (asterisks) GFP positive endocardial cells in a *Tg(flk:EGFP)* heart (B). Blends between the GFP and ISH signals (B,C,D’’). Inset (D’’) shows a *sema3fb*-expressing cardiomyocyte. **F-I** Whole mount (F-F’’,I) and sagittal (G-H) sections of *plxna3* ISH of 28 hpf (F-F’’,H–H’’), 36 hpf (G) and 48 hpf (I) zebrafish hearts. *plxna3* mRNA is present in GFP positive cardiomyocytes (arrows) in a *Tg(myl7:EGFP)* heart (F-F’,H–H’’) that surround the GFP positive endocardial cells (yellow arrowheads) in a *Tg(flk:EGFP)* heart (G). Blend between the GFP and ISH signals (F’’,G,H’’,I). A, anterior; a, atrium; e, eye; h, heart; hb, hindbrain; P, posterior; Tec, optic tectum; v, ventricle. Scale bar: 50 µm (A,C-D,F–H), 25 µm (B,E,I)

To elucidate the spatial and temporal expression of *sema3fb* in the zebrafish heart we performed whole mount RNA in situ hybridization (ISH) on embryos from 10–72 hpf. In zebrafish, cardiac progenitors of the atrial and ventricular chambers are bilaterally specified by 5 hpf [43]. The progenitors condense at the midline by 21 hpf and migrate to produce a linear tube of differentiating myocardium that surrounds the underlying endocardium [43, 44]. This tube starts pumping between 24–26 hpf [45]. By 48 hpf, the two-chambered heart is discernable by the constriction of the atrioventricular canal (AVC) [43, 46]. At 24 hpf, *sema3fb* mRNA was present through the linear heart tube (Fig. 1A). To confirm expression of *sema3fb* in cardiomyocytes, we cut JB4-plastic sections of *sema3fb* ISH on *Tg(flk:EGFP)* and *Tg(myl7:EGFP)* fish, where GFP labels the endocardial cells that line the ventricles and cardiomyocytes, respectively. Within the heart tube, *sema3fb* ISH signal was present adjacent to the GFP + endocardium (Fig. 1B) in cardiomyocytes throughout the *myl7:EGFP* heart tube, as visualized in whole mount (Fig. 1C,C’) and a sagittal section (Fig. 1D,D’,D’’). *sema3fb* mRNA remained in the ventricle and the atrium as the heart undergoes rightward looping and chamber morphogenesis (24–48 hpf), with expression at 48 hpf highest at the atrioventricular border (Fig. 1E, arrows). Weak expression persisted throughout the myocardium of the 72 hpf heart (data not shown). ISH of early stage embryos suggested that *sema3fb* mRNA was present in the bilateral heart fields at earlier stages, as marked by the cardiomyocyte progenitor marker *hand2* (Additional file 1: Fig. 1). These data indicate that mRNA for a secreted Sema is expressed by cardiomyocytes at the time they undergo differentiation, and agree with reported RNAseq analysis of FACs-isolated zebrafish cardiomyocytes, where *sema3fb* mRNA is enriched in the heart over the rest of the embryo [47].

Sema3s signal canonically through Nrp and Plxn receptors [13]. To identify potential receptors via which Sema3fb could influence cardiac chamber development we performed whole mount ISH for *plxn* and *nrp* genes. Expression of zebrafish *nrp* genes was reported previously [48–50]. *nrp2b* and *plxna3* mRNAs were present alongside *sema3fb* mRNA in the bilateral heart field at 18 hpf (Additional file 1: Fig. 2A-C), with mRNAs expressed at 28 hpf within the presumptive myocardium (Additional file 1: Fig. 2D,D’, Fig. 1F’). By 48 hpf, *nrp2b* and *plxna3* mRNAs were present in the ventricle and the anterior pole of the atrium (Fig. 1I, arrow; Additional file 1: Fig. 2F). Of note, other *plxna* genes were not expressed at significant levels in the 24 hpf heart (Additional file 1: Fig. 2G-J). To identify which heart cells express *plxna3* and *nrp2b* we performed whole mount ISH on *Tg(flk:EGFP)* and *Tg(myl7:EGFP)* fish at 28 hpf: Endothelial cells and cardiomyocytes are EGFP positive, respectively. For both *nrp2b* (Additional file 1: Fig. 2D,D’,E-E’’) and *plxna3* (Fig. 1F-F’’,H–H’’), ISH label was in GFP + cardiomyocytes, and not in the endocardium (Fig. 1G; data not shown). In summary, mRNAs for both Sema3fb and its known receptors are expressed by newly formed cardiomyocytes, arguing that Sema3fb plays a role in cardiomyocyte development. Interestingly, while *sema3fb* is expressed throughout the embryonic ventricle and atrial myocardium, the receptor data at 48 hpf suggest that Sema3fb signaling becomes spatially restricted mainly to the ventricular myocardium.

**Fig. 2:**
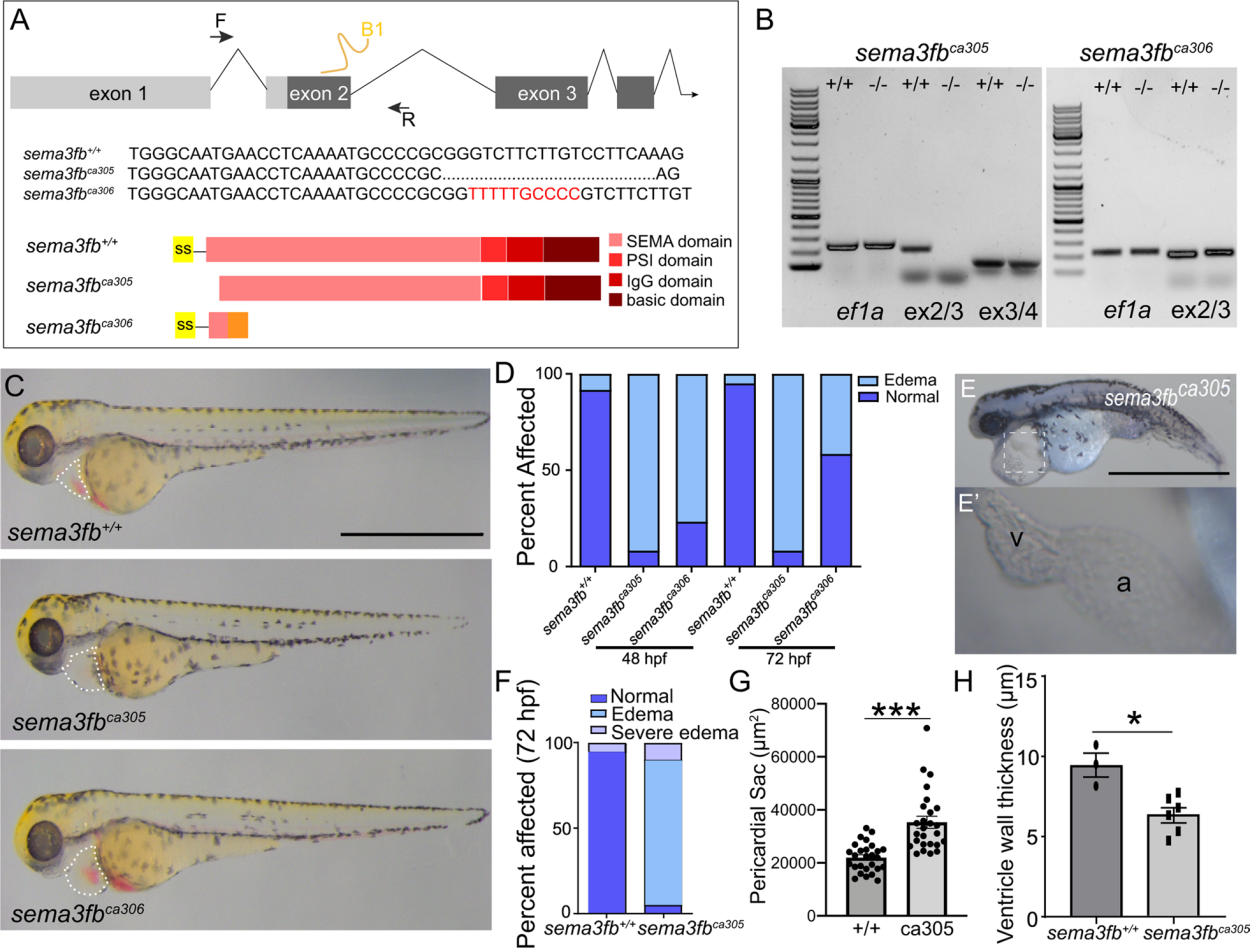
*sema3fb* mutants have cardiac edema. **A** Overview of the *sema3fb* locus targeted by CRISPR/Cas9 mutagenesis to exon 2 (guide RNA: B1) and primers used to identify the mutation. *sema3fb*^*ca305*^ fish have a 19 bp deletion (dots) and *sema3fb*^*ca306*^ fish have a 10 bp insertion (red sequence). Schematic representation of WT and mutant proteins. In the *sema3fb*^*ca305*^ allele, exon 1 is spliced in frame with exon 3, with loss of 62 amino acids (aa) at the beginning of a protein that would be translated, including the signal sequence (ss) and the first 14 aa of the SEMA domain. In the *sema3fb*^*ca306*^ allele, the 10 bp insertion introduces a frame shift and a premature stop codon to generate a predicted truncated 58 aa protein. F, forward primer; R, reverse primer. **B** RT-PCR indicates that exon 2 is skipped in the *sema3fb*^*ca305*^ allele; no PCR product is generated with exon 2-exon 3 primers, but product is generated with exon 3-exon 4 primers. Exon 2-exon 3 primers reveal exon 2 is present in the *sema3fb*^*ca306*^ allele. **C** Brightfield images of *sema3fb*^+/+^, *sema3fb*^*ca305*^ and *sema3fb*^*ca306*^ 72 hpf embryos. Dotted line outlines the pericardial sac. **D** The percent of embryos that present with cardiac edema at 48 hpf (N = 3, n = 60 for each genotype) and 72 hpf (N = 3, n = 60 for each genotype). **E** The severe mutant phenotype displaying major cardiac edema and failure to thrive (E), and the abnormal linear arrangement of the heart (E’, inset in E). **F** Quantitation of the severe edema phenotype at 72 hpf (N = 3, n = 180 per genotype). **G** Quantitation of the area of the pericardial sac (N = 2; WT n = 27; *sema3fb*^*ca305*^ n = 26, p < 0.0001). **H** The average ventricle wall width (µm) in 72 hpf *sema3fb*^*ca305*^ (n = 6) embryos is significantly thinner (*p* = 0.024) than that of WT (n = 3). Scale bar in C and E is 1 mm

### Generation of a Sema3fb loss-of-function genetic model

To assess the importance of Sema3fb for heart development and function we used CRISPR/Cas9 gene editing technology [32] to generate *sema3fb* genetic mutants. A specific guide RNA (sgRNA) targeting exon 2 (Fig. 2A) was co-injected with *cas9* mRNA at the one cell stage and a single founder was recovered that housed two allelic variants; a 19-base pair (bp) deletion and a 10-bp insertion. Both lines were propagated from this founder to generate stable homozygous viable generations, alongside wild type (WT) siblings. RT-PCR analysis of mRNA isolated from 24 hpf embryos indicated that the 19 bp deletion mutant (hereafter *sema3fb*^*ca305*^) lacked exon 2 that contains the ATG start site (Fig. 2B), presumably because the deletion at the end of exon 2 disrupts a key splice site. Indeed, sequencing of the PCR product generated with exon 1-exon 3 primers showed exon 1 spliced in frame with exon 3 to give an mRNA that if translated would produce a protein missing the first 62 amino acids (aa), including the signal sequence (amino acids 1–23) required for protein secretion and the first 14 aa of the SEMA domain necessary to elicit intracellular signaling [51] (Fig. 2A). Software could not identify another signal sequence in the predicted translated protein, thus in the *sema3fb*^*ca305*^ allele a C-terminal truncated Sema3fb, if even produced, would not be secreted from cardiomyocytes. RT-PCR and sequencing analysis indicated that exon 2 was included in the mRNA product in the 10 bp insertion allele (hereafter *sema3fb*^*ca306*^) (Fig. 2B). The *sema3fb*^*ca306*^ allele is predicted to produce a protein that is 58 aa in length and is prematurely truncated within the SEMA domain (Fig. 2A). Of note, a commercial antibody against mouse SEMA3F failed to distinguish between multiple Sema3s in zebrafish (data not shown). Importantly, injection of an antisense morpholino oligonucleotide (MO) against the ATG start site of *sema3fb* produced a similar heart edema when injected at the one-cell stage in WT embryos as seen for *sema3fb*^*ca305*^ (Additional file 1: Fig. 3A). MO injection into *sema3fb*^*ca305*^ embryos produced no greater incidence of severe edema (Additional file 1: Fig. 3A), arguing strongly for the specificity of the heart phenotype in *sema3fb* mutants being due to Sema3fb loss. Of note, *sema3fa* mRNA was not detected in the 48 hpf WT and *sema3fb*^*ca305*^ hearts (Additional file 1: Fig. 3B), indicating that Sema3fa upregulation does not compensate for the loss of Sema3fb.

**Fig. 3:**
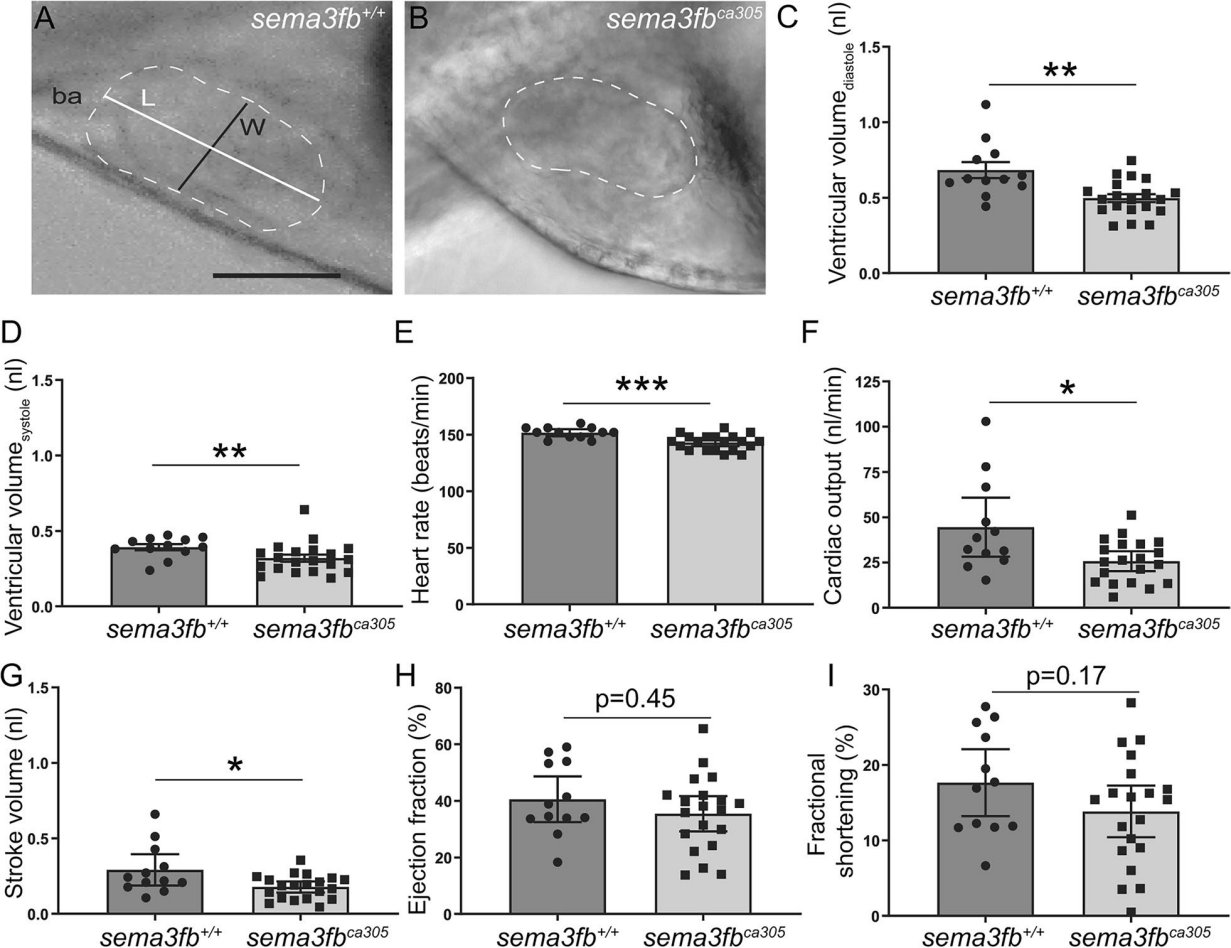
*sema3fb* mutant embryos have reduced cardiac function. **A, B** Still images from videos of beating WT and *sema3fb*^*ca305*^ mutant hearts at 72 hpf. Ventricle measurements were made in both the long axis (white line, L: length) and short axis (black line, W: width) to determine ventricle volumes. ba: bulbus arteriosus. **C, D** Quantitation of the average ventricle volume at end diastole (C) and end systole (D) reveals a significantly decreased capacity in the *sema3fb* mutant (*p* = 0.0027 and *p* = 0.0063, respectively) as compared to WT. **E-I** Graphs showing average heart rate (*p* = 0.0008) (E), cardiac output (*p* = 0.026) (F), stroke volume (*p* = 0.033) (G), ejection fraction (*p* = 0.45) (H), and fractional shortening (*p* = 0.17) (I). (N = 3; n = 12 *sema3fb*^+/+^, n = 20 *sema3fb*^*ca305*^). Scale Bar: 100 µm

### Embryos that lack Sema3fb exhibit cardiac edema

To examine potential heart defects with the loss of Sema3fb, heterozygous adults were incrossed, and progeny raised to homozygosity and incrossed with their respective genotypes (*sema3fb*^+*/*+^*, sema3fb*^*ca305*^*, sema3fb*^*ca306*^). 72 hpf embryos were indistinguishable by gross size, but homozygous embryos exhibited edema (Fig. 2C) that presented in both allelic variants at 48 hpf (8.3% *sema3fb*^+/+^, 91.7% *sema3fb*^*ca305*^, 76.7% *sema3fb*^*ca306*^, N = 3, n = 60 each genotype) and 72 hpf (5.0% *sema3fb*^+/+^, 91.7% *sema3fb*^*ca305*^, 41.7% *sema3fb*^*ca306*^, N = 3, n = 60 each genotype) (Fig. 2D). Most mutant embryos exhibited edema, though the severity of the defect varied. Of note, other zebrafish mutants show a similar variability in heart phenotype, with fish showing less severe edema able to recover and survive [52]. A small percentage of homozygous embryos exhibited severe cardiac edema and a hypoplastic ventricle and a linear arrangement of the heart tube (Fig. 2E,E’), and did not survive past 5–7 days post fertilization (6.1%, n = 82 embryos), (Fig. 2E,F). The remaining embryos developed to adulthood and were fertile. To quantitate the edema phenotype, we measured the area of the pericardial sac at 48 hpf: The area of the sac of *sema3fb*^*ca305*^ hearts (35,290 ± 2,251 µm^2^, n = 26 (standard error of the mean (SEM) here and throughout the paper, unless otherwise indicated), n = 27, p < 0.0001, Mann Whitney) was larger significantly than that observed in WT (21,983 ± 1,038 µm^2^) (Fig. 2G). Heterozygous *sema3fb* ± embryos also exhibited a spectrum of heart edema, ranging from no edema to severe edema, suggesting that genetic dosage influences the phenotype (data not shown). Of note, we excluded embryos with severe edema from further analysis so as not to confound data interpretation. As the phenotypes between the two allelic variants were highly comparable, we used *sema3fb*^*ca305*^ fish for our analyses, confirming key findings in *sema3fb*^*ca306*^ fish. Hematoxylin and eosin (H&E)-stained sections revealed that the thickness of the ventricle myocardium at 72 hpf was reduced significantly (6.3 ± 0.5 µm; Mann Whitney, *p* = 0.024, N = 2, n = 6) in *sema3fb*^*ca305*^ as compared to WT (9.4 ± 0.7 µm; N = 2, n = 3) hearts (Fig. 2H), suggesting that myocardial development was impaired.

### sema3fb mutants have impaired cardiac output and edema

By 72 hpf, cardiac function is considered rhythmic and the zebrafish circulatory system relatively mature [53]. To determine whether edema in *sema3fb*^*ca305*^ embryos resulted from impaired cardiac function, 72 hpf hearts were imaged by live video microscopy and ventricle measurements made at end-diastole and end-systole (Fig. 3A, B). Ventricle volume was reduced significantly during diastole (0.50 ± 0.03 nL; *p* = 0.0027) and systole (0.32 ± 0.02 nL; Mann Whitney, *p* = 0.0063) in *sema3fb*^*ca305*^ (N = 3, n = 20) as compared to WT (diastole, 0.68 ± 0.05 nL; systole, 0.39 ± 0.02 nL; N = 3, n = 12) siblings (Fig. 3C, D). Additionally, mutants had a small but significantly slowed heart rate (WT 151.7 ± 1.4 beats/min, N = 3, n = 12; *sema3fb*^*ca305*^ 143.0 ± 1.5 beats/min, N = 3, n = 20; Mann Whitney, *p* = 0.0008) (Fig. 3e). Cardiac output (WT 44.5 ± 7.4 nL/min, N = 3, n = 12; *sema3fb*^*ca305*^ 25.7 ± 2.6 nL/min; Mann Whitney, *p* = 0.021) and stroke volume (WT 0.29 ± 0.05 nL, N = 3, n = 12; *sema3fb*^*ca305*^ 0.18 ± 0.02 nL, N = 3, n = 21; Mann Whitney, *p* = 0.04) (Fig. 3F, G) were also reduced significantly in mutants as compared to WT, while ejection fraction (WT 40.5 ± 3.7%, N = 3, n = 12; *sema3fb*^*ca305*^ 35.4 ± 3.0%, N = 3, n = 20; Mann Whitney, *p* = 0.45) and fractional shortening (WT 17.6 ± 2.0%, N = 3, n = 12; *sema3fb*^*ca305*^, 13.8 ± 1.6%, N = 3, n = 20; Mann Whitney, *p* = 0.17) (Fig. 3H, I) were unchanged. Overall, the *sema3fb* mutant heart was impaired significantly, though parameters related to contractile function appeared preserved.

### Early heart development occurs normally in the sema3fb mutants

The edema of the *sema3fb* mutants suggested a defect in heart development. To begin to address the underlying biology, we examined whether early cardiac development was defective, since *sema3fb* appears to be expressed in the bi-lateral heart fields (Additional file 1: Fig. 1). Expression of *fgf8a*, a heart field marker [54] (Additional file 1: Fig. 3C-E), and cardiomyocyte progenitor markers *nkx2*.5 and *tbx5a* [55, 56] (Additional file 1: Fig. 3I-K), were not affected in the *sema3fb* mutants. Note that *fgf8a* expression was not impacted elsewhere in the embryo, including in the brain, somites and tailbud region (Additional file 1: Fig. 3F-H). Further, the expression of *myosin heavy chain 7* (*myh7*), a marker of ventricular cardiomyocytes [57], was condensed at the midline in similar numbers of WT (N = 2; n = 14/16) and *sema3fb*^*ca305*^ (n = 12/14) 21 hpf embryos (data not shown). These data suggest that the migration of cardiomyocytes to the midline and fusion to form the cardiac disc, and initial differentiation of ventricular cardiomyocytes, were not impacted by Sema3fb loss.

Next, we assessed the heart at 24 hpf by whole mount ISH for the cardiomyocyte marker *cardiac troponin T type 2a* (*tnnt2a*) [58], and for *myh7* and *myosin heavy chain 6* (*myh6*) [59] to label ventricular and atrial cardiomyocytes, respectively (Additional file 1: Fig. 4). These genes were present in both WT and mutant hearts. While 24 hpf WT hearts had elongated into linear tubes [60, 61] (Additional file 1: Fig. 4A,D,E,H,L), *sema3fb*^*ca305*^ and *sema3fb*^*ca306*^ hearts were still in a cone/condensed state (Additional file 1: Fig. 4B-D,F–H,L). The delay in heart morphogenesis was minimal as within two hours *sema3fb* mutant hearts elongated; 100% of *sema3fb*^*ca305*^ (N = 1, n = 7/7) and 90% of *sema3fb*^*ca306*^ (N = 1, n = 9/10) hearts (Additional file 1: Fig. 4N,O), comparable to the numbers observed in WT siblings (N = 1, n = 4/4) at 23 hpf (Additional file 1: Fig. 4M,P). Notably, the cranial ganglia that emerge immediately adjacent to the heart developed normally in aged-matched embryos (Additional file 1: Fig. 4Q,R). Overall, these data argue for largely normal initial formation of the atrial and ventricular chambers in the absence of Sema3fb.

**Fig. 4:**
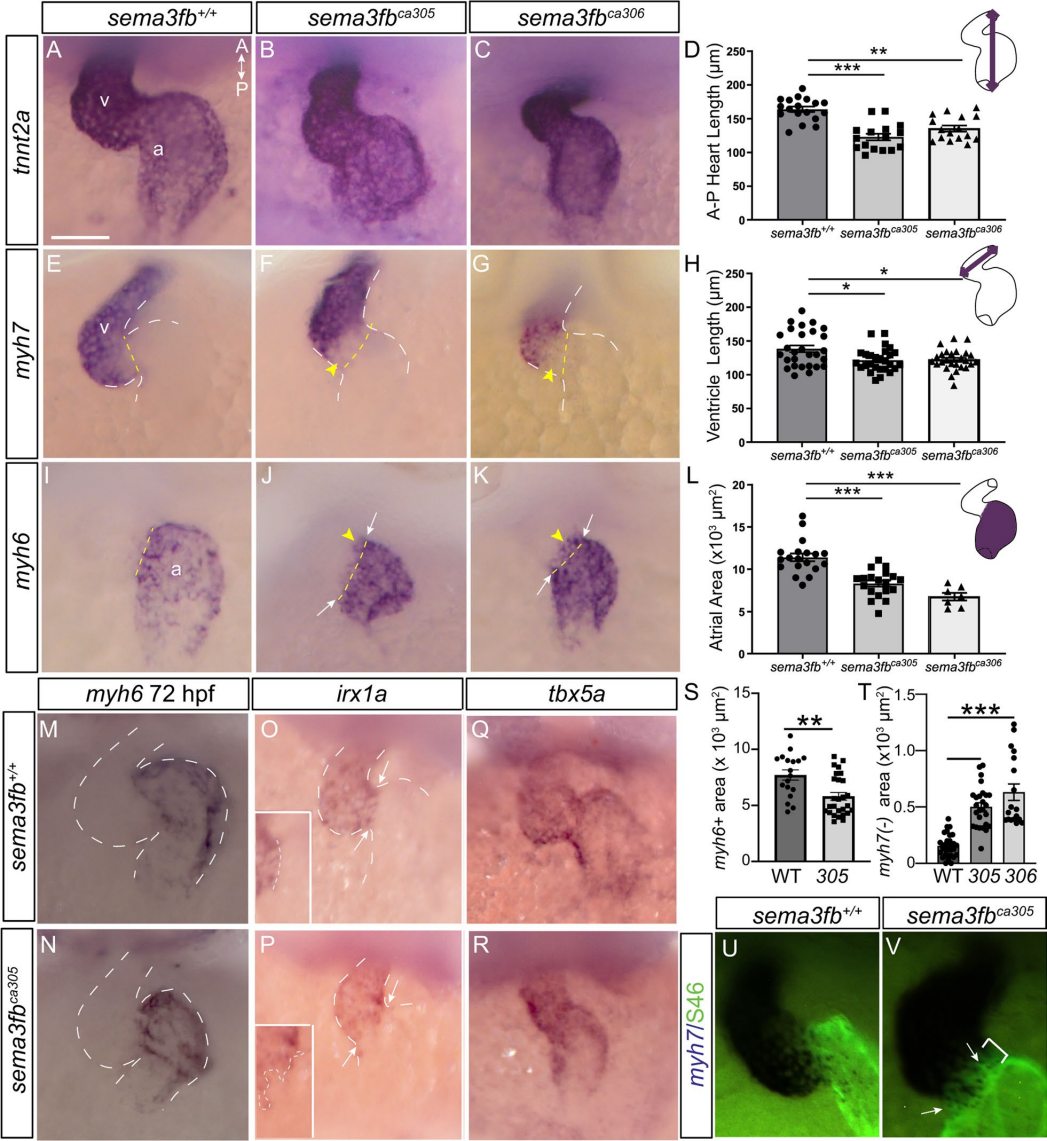
Ventricle and atrium are smaller in *sema3fb* mutant embryos. Whole mount ISH of 48 hpf hearts. Dotted yellow lines mark the presumptive border between the atria and ventricle placed at the morphological atrioventricular constriction. **A-C** The cardiomyocyte marker *tnnt2a* reveals large chambers in the WT heart (N = 2, n = 16/18) (A), while both *sema3fb* mutant alleles present with small hearts (B, N = 2, 14/16; C, N = 2, 15/16). **D** Antero-posterior heart length (purple line, schematic) reveals smaller *sema3fb*^*ca305*^ (p < 0.0001) and *sema3fb*^*ca306*^ (*p* = 0.0016) hearts as compared to WT. **E–G** Mutant alleles (F,G) present with smaller *myh7* + ventricles (N = 2, 17/20 *sema3fb*^*ca305*^; N = 2, 15/18 *sema3fb*^*ca306*^) than WT (N = 2, n = 19/20) (E), with loss of *myh7* label at the AVC (yellow arrowheads). **H** Shorter ventricles (purple line, schematic) in *sema3fb*^*ca305*^ (*p* = 0.009) and *sema3fb*^*ca306*^ (*p* = 0.013) as compared to WT embryos. **I-K** Mutant alleles present with smaller *myh6* + atria (N = 2, 18/21 *sema3fb*^*ca305*^; N = 2, 15/17 *sema3fb*^*ca306*^) (J,K) than in WT (N = 2, n = 19/19) (I), and ectopic *myh6* label in the ventricles (yellow arrowheads). **L** Quantitation of area (purple fill) reveals smaller atria in *sema3fb*^*ca305*^ (*p* = 0.0001) and *sema3fb*^*ca306*^ (p < 0.0001) hearts as compared to WT. **M, N** *myh6* expression at 72 hpf reveals a smaller *sema3fb*^*ca305*^ (N = 2, n = 27) atrium as compared to WT (n = 18). **O-R** Expression of *irx1a* (O-P; N = 2; WT n = 15/15; *sema3fb*^*ca305*^ n = 22/22) and *tbx5a* (Q-R; N = 2; WT n = 15/15; *sema3fb*^*ca305*^ n = 15/15) in the ventricle, with arrows marking the morphological atrioventricular constriction. Insets (O,P) reveal the *irx1a* border at the AVC, and the dotted lines the length of the *irx1a* domain border. **S** Mean area of *myh6* ISH label at 72 hpf (*p* = 0.0021). **T** Mean *myh7*(-) area measured between the morphological AVC and the posterior border of high *myh7* expression (p < 0.0001). **U, V** *myh7* ISH to mark ventricular cardiomyocytes and S46 antibody to label atrial cardiomyocytes. In the 48 hpf *sema3fb*^*ca305*^ heart there is a gap between the two labels (bar) into which S46 immunolabel extends (arrows). A: anterior; a, atrium; P: posterior; v, ventricle. Scale bar 50 µm

### Heart chamber size is decreased in sema3fb mutants

We next asked whether heart development proceeded normally after initial formation of the linear heart tube. We investigated *sema3fb*-/- hearts at 48 hpf, when heart chamber morphogenesis is complete [60]. We first used *tnnt2a* to label both atrial and ventricular cardiomyocytes. Looped hearts with ventricular and atrial chambers were present across all genotypes (Fig. 4A-C). Yet, the antero-posterior length of the *tnnt2a*-labelled heart was significantly shorter in both *sema3fb*^*ca305*^ (122.8 ± 5.0 µm, N = 3, n = 16; Kruskal–Wallis, Dunn’s multiple comparisons, p < 0.0001) and *sema3fb*^*ca306*^ (135.4 ± 4.5 µm, N = 3, n = 16; Kruskal–Wallis, *p* = 0.0016) embryos than WT (163.8 ± 4.0 µm, N = 3, n = 18) (Fig. 4D).

To determine if cardiac chambers were affected we used whole mount ISH at 48 hpf for *myh7* (Fig. 4E-G) and *myh6* (Fig. 4I-K) to label ventricular and atrial cardiomyocytes, respectively. Both cardiac chambers were smaller in mutants as compared to WT. The long axis of the *sema3fb*^*ca305*^ (121.5 ± 3.3 µm, N = 3, n = 27; Ordinary one-way ANOVA, Tukey’s multiple comparison test, *p* = 0.009) and *sema3fb*^*ca306*^ (122 ± 3.1 µm, N = 3, n = 26; Ordinary one-way ANOVA, *p* = 0.013) ventricles was significantly shorter than in WT (138.4 ± 5.1 µm, N = 3, n = 28) (Fig. 4H), as confirmed by measuring the ventricle length during diastole (WT 203.9 ± 6.7 µm, n = 12; *sema3fb*^*ca305*^ 190.5 ± 2.3 µm, n = 20; Mann Whitney, *p* = 0.048) and systole (WT 175.6 ± 4.2 µm, n = 12; *sema3fb*^*ca305*^ 163.3 ± 3.4 µm, n = 20; Mann Whitney, *p* = 0.031) in live embryos (Additional file 1: Fig. 5). Additionally, the areas of *myh6* + atria were significantly smaller in *sema3fb*^*ca305*^ (8,290 ± 360 µm^2^, N = 3, n = 19; Kruskal–Wallis, Dunn’s multiple comparisons, *p* = 0.0001) and *sema3fb*^*ca306*^ (6,760 ± 450 µm^2^; N = 2, n = 7 Kruskal–Wallis, Dunn’s multiple comparisons, p < 0.0001) hearts than in WT (11,390 ± 460 µm^2^ [2]; N = 3, n = 19) (Fig. 4I-L). The small *myh6* + atrial chamber was still present in *sema3fb* mutants at 72 hpf (N = 2; WT, 7,713 ± 454 µm^2^ [2], n = 18 embryos; *sema3fb*^*ca305*^ 5,815 ± 346 µm^2^ [2], n = 27; *p* = 0.0021 Mann Whitney) (Fig. 4M, N, S). Ultimately, these data suggest that with the loss of Sema3fb both ventricle and atrium are specified, but the chambers are smaller in size than seen in WT.

**Fig. 5:**
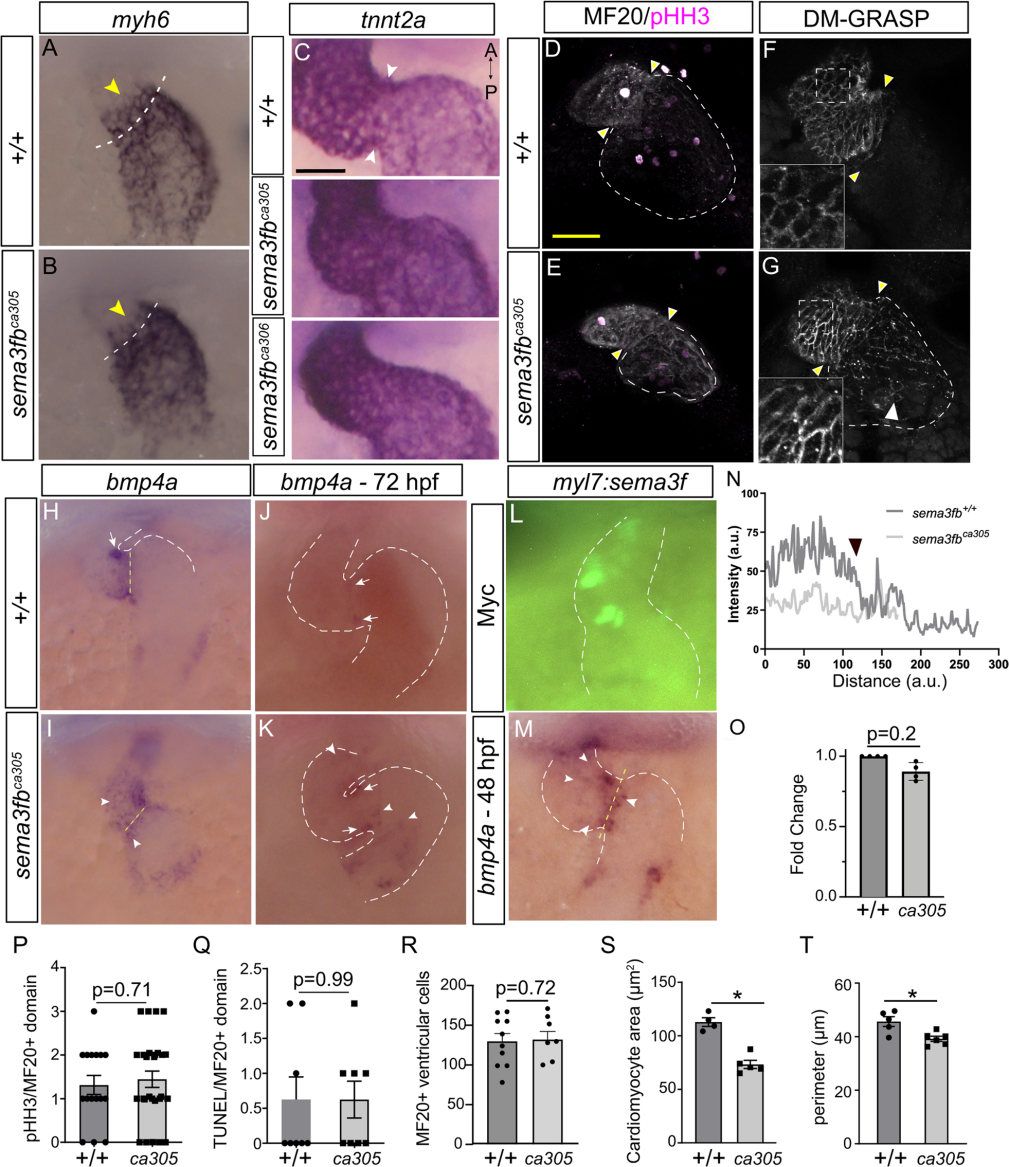
Chamber identity disrupted at the AVC with loss and overexpression of Sema3f. **A**, **B** *myh6* in 36 hpf mutant hearts expressed anterior (arrowheads) to the AVC (dotted line). **C** Discrete change in *tnnt2a* ISH signal at the atrial-ventricular border in WT but not mutant 48 hpf heart (arrowheads). **D-G** Confocal of 48 hpf hearts immunolabeled for pHH3 and MF20 (D,E), and DM-GRASP (F,G). Discrete expression border at the AVC (yellow arrowheads) in WT (D, N = 1, n = 5/5, and F, N = 3, n = 11/12) but not mutant hearts (E, N = 1, n = 8/9 and G (white arrowhead), N = 3, n = 10/12). **H–K** *bmp4a* mRNA restriction to the AVC (arrows) in WT at 48 hpf (H, N = 4, n = 21/30) and 72 hpf (J, N = 1, n = 8/9), but expansion beyond the AVC (arrowheads) in mutants (I, 48 hpf, n = 36/45; K, 72 hpf, n = 10/11). **L, M** *myl7:sema3f* plasmid-driven Sema3f expression in heart cardiomyocytes, with immunolabeling for myc-tagged Sema3f (L) and ISH for *bmp4a* (M) at 48 hpf. *bmp4a* mRNA expands beyond the AVC with Sema3f overexpression (arrowheads, M; N = 2, n = 24/37 expanded) but not control (*bmp4a* n = 17/20). **N** Average RGB MF-20 immunolabel intensity across the antero-posterior heart axis. High expression in WT (n = 5) ventricle sharply tapering at the atrial border (~ 150 arbitrary units (a.u.); arrowhead) is not seen in mutants (n = 9). Note *sema3fb* mutant hearts are small. **O** RT-qPCR for *bmp4a* mRNA (N = 4, *p* = 0.68). **P-R** Mean pHH3 + actively dividing MF20 + cardiomyocytes (P, *p* = 0.71), TUNEL + /MF20 + cardiomyocytes (Q, *p* = 0.99), and MF20 + ventricular cardiomyocytes (R, *p* = 0.72) at 48 hpf. **S, T** Mean ventricular cardiomyocyte area (S) and perimeter (T) in *sema3fb*^*ca305*^ as compared to WT (*p* = 0.016 and *p* = 0.03, respectively). N = 3, n = 4–5 WT and n = 6 mutants, with 9–12 DM-GRASP + cells/embryo. Scale bar in C is 40 µm and in D is 25 µm (A,B,D-M)

We next assessed cardiomyocyte differentiation, focusing on the ventricle, as at 48 hpf cardiomyocytes in this chamber express the requisite receptors to respond to Sema3fb. We assessed the expression of the ventricle-specific homeobox-containing transcription factor, *iroquois1a* (*irx1a*) [62], and *T-box transcription factor* (*tbx5a*), which at 48 hpf is expressed in the ventricle and anterior atrium [63]. *irx1a* was expressed in both the WT and mutant ventricle (Fig. 4O, P; N = 2; WT, n = 15/15 hearts; *sema3fb*^*ca305*^ n = 22/22), at comparable levels as measured by RT-qPCR (1.2 ± 0.4 (standard deviation) fold change vs. WT, N = 4; Mann Whitney, *p* = 0.69). *tbx5a* was also expressed in WT (N = 1, n = 9) and *sema3fb*^*ca305*^ (N = 1, n = 7) ventricles (Fig. 4Q, R), apparently at similar levels as quantitated by RT-qPCR (-/- 0.93 ± 0.3 (standard deviation) fold change vs. WT, N = 3; Mann Whitney, *p* = 0.7). These data indicate that while the *sema3fb*^*ca305*^ ventricle is smaller than in WT, the ventricular differentiation program is not affected globally by Sema3fb loss.

### The border between the ventricle and atrium appears disrupted with loss of Sema3fb

The fact that *sema3fb* mRNA is expressed by cardiomyocytes of both atrial and ventricular chambers, while key receptors are present predominantly in the 48 hpf ventricle, combined with the known role for Sema3s in providing positional information to cells [64], raised the possibility that Sema3fb might provide spatial information to cardiomyocytes as to their ventricular localization. In this respect, we were intrigued by an apparent disruption of the tight border between the expression of atrial vs. ventricular chamber specific genes in 48 hpf mutant hearts. In most WT hearts (N = 5, 30/31 hearts), the *myh7*-expression border aligned with the morphological constriction between the ventricle and atrial chambers (dotted line, Fig. 4E). In contrast, in both *sema3fb*^*ca305*^ (N = 4, n = 21/26 hearts; Fig. 4F) and *sema3fb*^*ca306*^ (N = 3, n = 18/18; Fig. 4G) hearts, the posterior border of the high *myh7* expression domain sat anterior to the constriction, with *myh7* ISH signal low or absent from the atrioventricular band (yellow arrowheads, Fig. 4F, G). To quantitate this phenotype, we measured the area between the atrio-ventricular constriction and the border of high *myh7* expression. This area was quite small in most WT hearts, reflecting the alignment of ventricular *myh7* expression with the atrioventricular constriction (WT 156.6 ± 18 µm^2^, n = 31 embryos) (Fig. 4T). Because the *myh7* border often sat anterior to this morphological constriction in mutants, the *myh7*(-) area was significantly larger than in WT (*sema3fb*^*ca305*^ 502.1 ± 36 µm^2^ n = 26; *sema3fb*^*ca306*^ 631.7 ± 7 µm^2^, n = 18; p < 0.0001 One Way Ordinary ANOVA, Dunnett’s multiple comparisons).

The *myh6* expression border between the atrium and ventricle was also disrupted in mutant hearts. In most WT hearts the anterior border of *myh6* expression aligned with the morphological constriction between the atrium and ventricle (n = 20/26), but expression often extended a small extent past this border into the ventricle in *sema3fb*^*ca305*^ (N = 4, n = 23/31) and *sema3fb*^*ca306*^ (N = 1, n = 6/7) hearts (Fig. 4I-K; yellow arrowheads). To visualize the border between the ventricle and atrium we marked ventricular cardiomyocytes by *myh7* ISH and atrial cardiomyocytes by S46 immunolabeling [65]. Here it was obvious that while the two markers abutted each other in the WT heart (Fig. 4U; n = 6/6), they were separated from one another in the mutant heart and S46 immunoreactivity extended into the gap (arrows, Fig. 4V; n = 6/6 separated). To ask whether border defects were present earlier in *sema3fb* mutant hearts we assayed *myh6* at 36 hpf. Interestingly, *myh6* label anterior to the atrioventricular constriction was present in both the WT and *sema3fb*^*ca305*^ heart (Fig. 5a, b). These data suggest that in WT an initially sloppy border resolves over time as the chambers continue to mature, a process that fails to occur in the absence of Sema3fb.

To further investigate the chamber border, we assessed other markers that show differential expression between the atrium and ventricle. In WT hearts, the border of the *irx1a* at the AVC is straight, but was imprecise in *sema3fb*^*ca305*^ hearts, as evident by an increase in the length of the ISH border (Fig. 4O, P insets, dotted lines) in mutants vs. WT hearts (N = 2, WT 44.3 ± 3 µm, n = 13; *sema3fb*^*ca305*^ 85.3 ± 9 µm, n = 21; p < 0.0001, Mann Whitney). In support, *tnnt2a* mRNA exhibited a sharp drop in level in the atrium relative to the ventricle in WT hearts (Fig. 5C), but lacked an obvious change in expression between the two chambers in both mutant alleles. To confirm at the protein level that there was disruption of the chamber border in *sema3fb* mutants at 48 hpf, we used antibodies for proteins expressed at higher levels by ventricular than atrial myocardium: MF20 is a marker in zebrafish that appears more robustly expressed by ventricular than atrial cardiomyocytes [66], and DM-GRASP marks the surface of cardiomyocytes [67]. Whole mount immunostaining, followed by confocal microscopy, revealed separate atria and ventricles in WT hearts at 48 hpf (N = 1, n = 5/5 MF20; N = 3, n = 11/12 DM-GRASP, Fig. 5D, F). In contrast, the distinct expression border between the chambers was disrupted in at least 83% of mutants (N = 1, n = 8/9 MF20; N = 3, n = 10/12 DM-GRASP, Fig. 5E, G). This loss of a sharp ventricle to atria border was evident with the quantitation of the average intensity of MF20 immunolabel over the antero-posterior axis of the heart (WT n = 5, *sema3fb*^*ca305*^ n = 9; Fig. 5N).

We next asked if altered gene expression at the atrioventricular border impacted *bone morphogenetic protein 4a* (*bmp4a*) mRNA, which is expressed initially throughout the antero-posterior length of the myocardium and restricts to specialized cardiomyocytes of the AVC by 37 hpf [68]. We found the strongest *bmp4a* mRNA signal was in the AVC region in almost all WT (N = 4; 96.7%, n = 29/30) and *sema3fb*^*ca305*^ (N = 4; 91%, n = 41/45) 48 hpf hearts (Fig. 5H, I). In most WT hearts, *bmp4a* was restricted to the AVC alone (N = 4; 70%, n = 21/30), with a small number of hearts showing additional ventricular (20% n = 6/30) or atrial (13.3%, n = 4/30) ISH signal (Fig. 5H). In contrast, *bmp4a* mRNA often continued to be expressed throughout the mutant ventricle (71.1%, n = 32/45) and/or the atrium closest to the AVC (53.3% n = 24/45) (Fig. 5I). Blinded analysis supported a significant increase in the area of the high *bmp4a* AVC expression measured from the whole mount ISH images in the mutant vs. WT heart (N = 4, WT 1770 ± 145 µm^2^, n = 30; 4109 ± 332 µm^2^, n = 45; p < 0.0001 Mann Whitney). While the *bmp4a* domain was altered in the *sema3fb*^*ca305*^ 48 hpf heart, this was not evident as an upregulation of *bmp4a* mRNA levels as measured by RT-qPCR (Fig. 5O; N = 4, 0.89 ± 0.06 fold change, *sema3fb*^*ca305*^ vs. WT. Error bars represent standard deviation. *p* = 0.68, Mann Whitney), though changes would be difficult to observe as *bmp4a* was expressed at high levels in both the inflow and outflow tracts of the mutant and WT heart. By 72 hpf, mutant hearts still exhibited a handful of *bmp4a*-expressing ectopic cells within the ventricle (60% n = 9/15) or atrium (73% n = 11/15), though *bmp4a* expression was concentrated mainly at the AVC, as was the case in all WT hearts (n = 24/24) (Fig. 5J,K).

The expansion of *bmp4a* expression in mutant hearts could have occurred because of a direct effect of *Sema3fb* on gene transcription; Sema3fb may normally inhibit *bmp4a* expression by ventricular cardiomyocytes that are not part of the AVC. If true, overexpression of Sema3f in ventricular cardiomyocytes might be expected to reduce *bmp4a* mRNA levels. To test this possibility, we injected a Tol2 *myl7:sema3f-myc* construct into embryos at the one-cell stage along with *transposase* mRNA, with injection of the plasmid alone serving as control. While we tried but failed to clone *sema3fb*, the fact that Sema3fa and Sema3fb paralogs share 83% amino acid identity, and *sema3fa* or *sema3fb* mRNA both result in delayed neutrophil recruitment in a zebrafish model of inflammation [69], argued that ectopic Sema3fa could substitute functionally for Sema3fb. Thus, we took advantage of an available full-length cDNA clone of zebrafish *sema3fa* for this assay. At 48 hpf, the *sema3f*-expressing fish were processed by whole mount ISH for *bmp4a* mRNA. Notably, Sema3f was expressed in a mosaic fashion in both the atrium and ventricle, as visualized by immunolabeling for the myc-tag of Sema3fa (Fig. 5L). Interestingly, Sema3f overexpression produced a similar *bmp4a* phenotype to that seen with loss of Sema3fb. Both control and Sema3f-expressing 48 hpf hearts exhibited enhanced expression of *bmp4a* at the AVC (Control, 100% n = 20/20; Sema3f overexpression, 97% n = 35/36). While *bmp4a* mRNA was present only within the AVC in most control hearts (75%, n = 15/20), this was true in a small number of *sema3f*-overexpressing hearts (25%, n = 9/36), where the *bmp4a* domain spread beyond the AVC into the atrium and/or ventricle (arrowheads, Fig. 5M). Measurement of the area of the *bmp4a* expression domain in WT and mutant hearts supports this observation (WT 1652 ± 244 µm^2^, n = 20; Sema3fa overexpression 3346 ± 205 µm^2^, n = 36; Mann–Whitney, p < 0.0001). These data argue that Sema3fb does not inhibit *bmp4a* transcription. The fact that both loss and gain of Sema3f function produced a similar phenotype argues that normally Sema3fb signaling needs to be strictly regulated, and that either too little or too much signaling disrupts normal development of the border between the ventricular and atrial chambers, such that that the regulation of *bmp4a* expression is disrupted.

### Cardiomyocyte size decreased in sema3fb mutants

The small chamber sizes in *sema3fb* mutant hearts at 48 hpf could be due either to fewer cardiomyocytes or a reduction in cardiomyocyte size. Cardiomyocyte progenitor cells are specified and differentiate in the absence of Sema3fb, but less proliferation and/or increased apoptosis could subsequently reduce their numbers. Yet, similar numbers of mitotically active cardiomyocytes were labelled with an antibody against phosphorylated histone H3 (pHH3) in 48 hpf WT and *sema3fb*^*ca305*^ hearts (WT 1.31 ± 0.2 cells, N = 3, n = 16; *sema3fb*^*ca305*^ 1.44 ± 0.2 cells, N = 3, n = 27; Mann Whitney, *p* = 0.71) (Fig. 5D, E, P). Furthermore, at 48 hpf the two genotypes exhibited similar numbers of TUNEL + apoptotic cardiomyocytes (WT 0.62 ± 0.3 cells, N = 2, n = 8; *sema3fb*^*ca305*^ 0.62 ± 0.3 cells, N = 2, n = 8; Mann Whitney, p > 0.99) (Fig. 5Q). and MF20 + ventricular cells (WT 129.7 ± 10, n = 10; 132 ± 10, n = 7; *p* = 0.72, Mann Whitney) (Fig. 5R). Finally, ISH at 48 hpf with a marker of cardiac neural crest (CNC), *cysteine-rich intestinal protein 2* (*crip2*) (N = 1, n = 11 WT and N = 1, n = 11 *sema3fb*^*ca305*^) [70], and of cells of the secondary heart field (*latent tgfβ binding protein 3a* (*ltbp3a*)) (N = 1, n = 6 WT and N = 1, n = 7 *sema3fb*^*ca305*^) [56, 71], showed that these two sources provided cardiomyocytes for the WT and mutant hearts (data not shown). These data argue that a reduction in cardiomyocyte number does not contribute substantially to the smallness of the *sema3fb*-/- heart.

Cardiomyocytes normally enlarge as the zebrafish cardiac chambers emerge (36 hpf), and between 40–45 hpf more than half of ventricular cardiomyocytes increase in size [67, 72]. Thus, we asked whether cardiomyocyte size was impacted in the mutants. We focused on ventricular cardiomyocytes, because we found that DM-GRASP more effectively labelled the lateral edges of the ventricular than atrial cardiomyocytes (Fig. 5F). Ventricular cardiomyocyte size was obviously different between the WT and *sema3fb*^*ca305*^ embryos (insets, Fig. 5F, G). To quantitate this difference, we took confocal projections of DM-GRASP-labeled hearts and measured area, perimeter and circularity of cardiomyocytes within the central region of the ventricle. Circularity was comparable across WT and mutant cardiomyocytes (WT 0.66 ± 0.02, n = 5 embryos (9–12 cells measured/embryo); *sema3fb*^*ca305*^ 0.63 ± 0.03, n = 6 embryos; Mann Whitney, *p* = 0.66), however, both ventricular cardiomyocyte area (WT 112.8 ± 4.0 µm^2^, n = 4 embryos; *sema3fb*^*ca305*^ 73.3 ± 3.8 µm^2^, n = 5 embryos; Mann Whitney, *p* = 0.016, Fig. 5S) and perimeter (WT 45.6 ± 1.8 µm, n = 5 embryos; *sema3fb*^*ca305*^ 39.2 ± 1.0 µm, n = 6 embryos; Mann Whitney, *p* = 0.03, Fig. 5T) were reduced in the mutant embryos. These data suggest that reduced cardiomyocyte size explains in part the small heart chambers of mutants.

Because cardiomyocyte size is linked intimately to the hemodynamic forces of flow within the heart [67, 72], we asked whether *sema3fb* mutant hearts were still smaller than their WT counterparts when cardiac flow was removed from the equation by growing hearts independent of the circulatory system. WT and mutant hearts were explanted at 24 hpf, cultured for 24 h, and assessed by ISH for the expression of atrial *myh6* (Additional file 1: Fig. 6A, B). *myh6* + mutant atria were significantly smaller (N = 2 WT 9,982 ± 490 µm^2^, n = 10 explants; *sema3fb*^*ca305*^ 6,035 ± 633 µm^2^, n = 13 explants; Mann Whitney, *p* = 0.0011) in size as compared to WT (Additional file 1: Fig. 6C), arguing that chamber defects present in the Sema3fb mutants independently of hemodynamic forces.

**Fig. 6:**
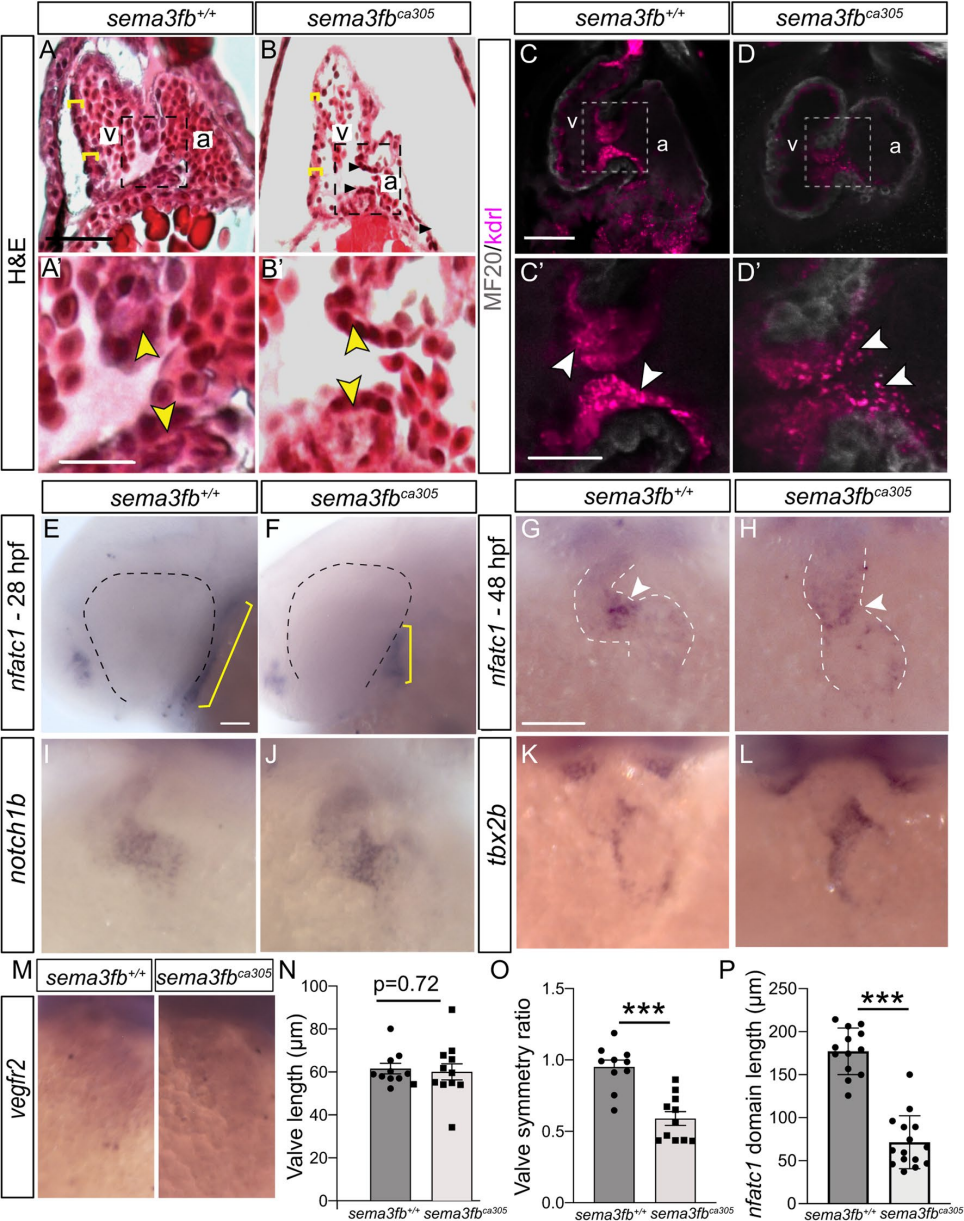
Disrupted heart valve development with the loss of Sema3fb. **A, B** Hematoxylin and eosin stained sections show the ventricle walls (yellow brackets) and valves (arrowheads within inset) of WT (A, A’) and *sema3fb*^*ca305*^ embryos (B, B’) at 72 hpf. **C**, **D** Confocal projections of 72 hpf WT (C, C’) and mutant (D,D’) hearts immunolabeled by the myocardial marker MF20 (grey) on an endocardial-labeled transgenic line *Tg(kdrl:mCherry)*. Brightness was increased in D’ to allow for comparison with C’. **E**, **F** *nfatc1* ISH label in WT (E; N = 2, n = 13/13 normal) and *sema3fb*^*ca305*^ (F, n = 13/15 short) 28 hpf embryos reveals a smaller endocardial domain (yellow bars). **G, H** *nfatc1* concentration at the AVC (arrowheads) in WT (G, n = 21/21) and mutant (H, n = 16/17) 48 hpf fish. **I-M** AVC endocardial marker *notch1b* (I,J) (N = 2 WT n = 10/10; *sema3fb*^*ca305*^ n = 10/10) and endocardial marker *vegfr2* (M) (WT n = 10/12; *sema3fb*^*ca305*^ n = 15/17) in 48 hpf hearts. Expansion of the *tbx2b* AVC cardiomyocyte marker in mutants (n = 7/8) that normally shows restricted expression at the AVC in WT hearts (n = 5/6) (K,L). **N-P** Quantitation of the: (1) length of the AV valve, as marked by mCherry in the *Tg(kdrl:mCherry)* background (N; N = 2, n = 10 *sema3fb*^+/+^, n = 12 *sema3fb*^*ca305*^), (2) the ratio of the length of the valve present in the atrium to that measured in the ventricle at 72 hpf (O; *p* = 0.0003), and (3) the length of the *nfatc1* domain (yellow bars, E and F) in 28 hpf WT (n = 13) and mutant (N = 15) embryos (P; p < 0.0001). Scale bars in A (A,B), A’ (A’,B’), C (C,D), C’ (C’,D’), E (E,F), G (G-M) are 50 µm

### Heart valve development disrupted slightly with Sema3fb loss

The defects in gene expression observed at the AVC of *sema3fb* mutants led us to ask if heart valves develop normally, as cells at the boundary between the two chambers give rise to the mature valves [46, 73]. Valves were present in *sema3fb*^*ca305*^ hearts when assessed in H&E-labeled 72 hpf sections (Fig. 6A, B). To confirm these data, we bred the *sema3fb*^*ca305*^ allele onto the *Tg(kdrl:mCherry)* line, in which the endocardial cells that make up the valves [73] are mCherry + at 72 hpf [29]. In both the WT and *sema3fb*^*ca305*^ hearts, valves were present at the border between the atrium and ventricle (Fig. 6C, D), and were of similar lengths (N = 2; WT 61.6 µm ± 2.5 µm, n = 10 embryos; *sema3fb*^*ca305*^ 60.1 µm ± 3.8 µm, n = 12 embryos; Mann Whitney, *p* = 0.72) (Fig. 6N). The intensity of the mCherry label in the *sema3fb*^*ca305*^ valves, however, was reduced as compared to WT (Fig. 6C, D). Further, the distribution of the valves differed between the genotypes, with the WT valves roughly symmetrically distributed on either side of the constriction between the atrial and ventricular chambers, but preferentially located on the ventricular side of this border in mutants (Fig. 6O).

To assess whether early development of the endocardium, from which valves form, occurred normally in the mutants, we examined the expression of the early endocardial marker *nfatc1* [74]. While *nfatc1* ISH signal was present in WT and mutant 28 hpf hearts, the domain in *sema3fb*^*ca305*^ embryos (71.4 µm ± 8 µm, N = 2, n = 15 embryos) was shorter than in WT (N = 2, 177.2 µm ± 8 µm, N = 2, n = 13; Mann Whitney, p < 0.0001) (Fig. 6E, F, P). Yet, mRNAs for *nfatc1* (N = 2, WT, n = 21/21 embryos; *sema3fb*^*ca305*^, n = 16/17 embryos; arrowheads, Fig. 6G, H) and an additional endothelial AVC marker, *notch1b* (N = 2, WT, n = 10/10 embryos; *sema3fb*^*ca305*^, n = 10/10 embryos; Fig. 6I, J), were localized preferentially to the AVC in both genotypes at 48 hpf. Further, the endothelial marker *vegfr2* was present in both chambers of WT and mutant hearts (Fig. 6M). Yet, the domain of the AVC cardiomyocyte marker, *tbx2b* (Fig. 6K, L) [75], while preferentially expressed at the AVC, appeared expanded in size in mutant vs. WT embryos at 48 hpf (WT, n = 5/6 embryos with normal *tbx2b*; *sema3fb*^*ca305*^, n = 7/8 with expanded *tbx2b*). In support, the area of the *tbx2b* domain in *sema3fb*^*ca305*^ hearts was significantly greater than in WT (N = 1, WT, 535.3 ± 132 µm^2^ [2], n = 6; *sema3fb*^*ca305*^, 1084 ± 139 µm^2^ , *p* = 0.02, Mann Whitney). These data suggest the slight delay in endocardial development, similar to what is seen with heart tube elongation (Additional file 1: Fig. 4), does not grossly impact the endothelial contribution to the newly formed AVC in 48 hpf *sema3fb*^*ca305*^ embryos. Yet, the organization of the *tbx2b* positive AVC cardiomyocytes is somewhat disrupted in the absence of Sema3fb.

### Plxna3 may mediate the Sema3fb signal

**Fig. 7:**
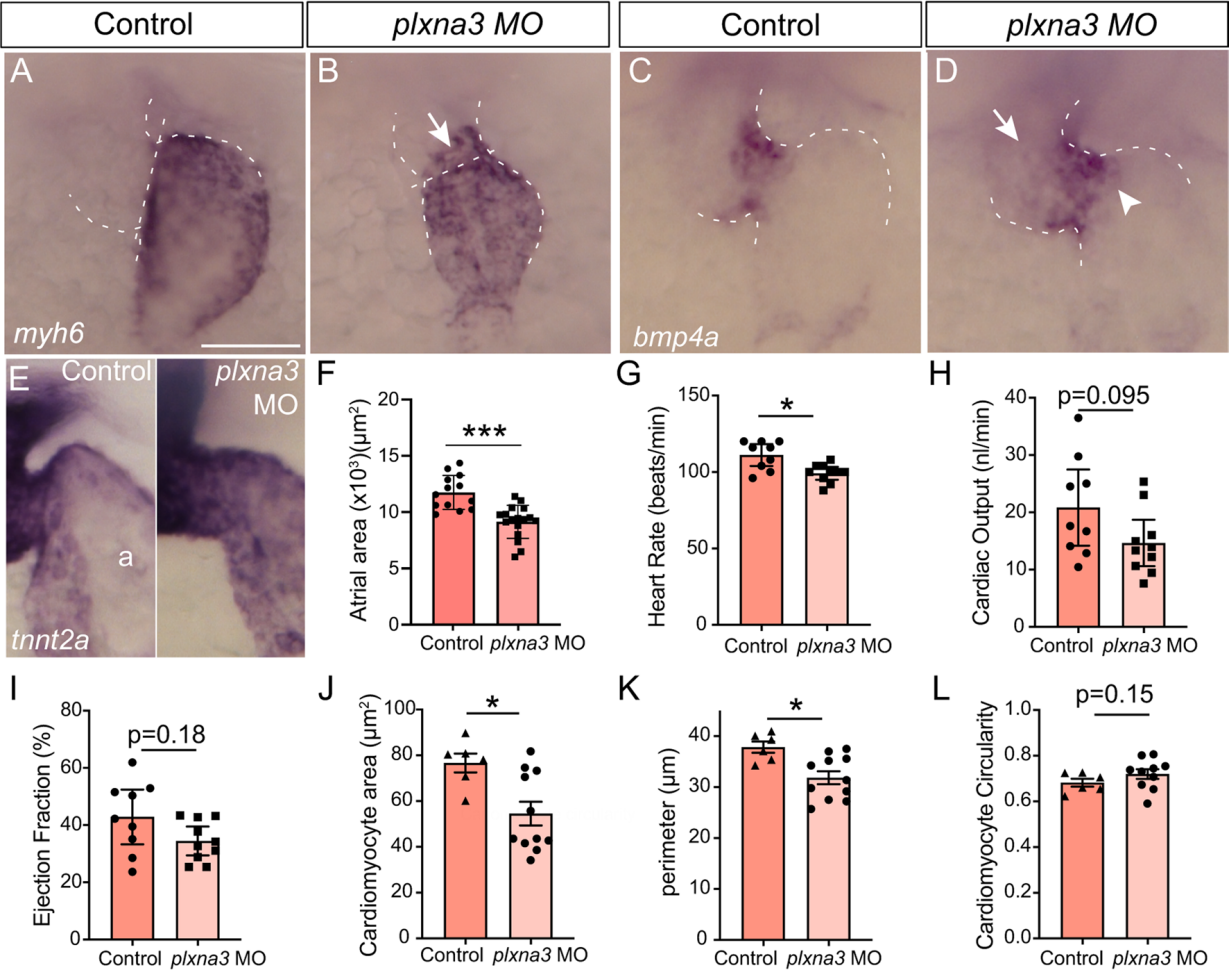
Plxna3 is a candidate receptor for Sema3fb. **A, B** *myh6*-expressing atria of control (A) and *plxna3* (B) morpholino-injected 48 hpf embryos (N = 1 WT n = 5/5 normal; *plxna3* MO, n = 9/15 small atria). White line indicates the morphological constriction between the atrium and ventricle, and arrow points to ectopic ISH label in the ventricle. **C**, **D** *bmp4a* expression in control (C) and *plxna3* MO (D) embryos. Ectopic ISH label in the ventricle (arrow) and atria (arrowhead). **E** *tnnt2a* shows a sharp reduction at the AVC in a control (n = 8/8) but not a *plxna3* MO (n = 7/10 imprecise) embryo. **F** Mean atrial area is reduced in *plxna3* MO embryos (N = 2; p < 0.0001, n = 17) as compared to controls (n = 13). **G-I** Average heart rate (*p* = 0.011, G), cardiac output (*p* = 0.095, H), and ejection fraction (*p* = 0.18, I) (N = 3, n = 9 controls, n = 10 *plxna3* MO). **J-L** Cell size of DM-GRASP + ventricular cardiomyocytes. A significant reduction is seen in cardiomyocyte area (J, *p* = 0.019) and perimeter (K, *p* = 0.015) in *plxna3* MO as compared to control embryos, but not circularity (L, *p* = 0.15). N = 3, n = 6 control and n = 11 *plxna3* MO embryos, with 8–10 cells/embryo. Scale bar in A is 50 µm

To identify which receptor mediates Sema3fb signaling, we asked whether loss of either of the two receptors expressed by ventricular cardiomyocytes, *nrp2b* and *plxna3*, recapitulated the *sema3fb* mutant heart phenotype. We performed MO-mediated knockdown of Plxna3 and Nrp2b with previously characterized MOs [42]. Previous analysis revealed heart edema in Nrp2b morphants [48], pointing to a role for Nrp2b in heart development. We found that knockdown of either receptor resulted in edema by 48 hpf, indicating that heart function was compromised. The hearts of *nrp2b*-deficient embryos were grossly malformed, with a severe reduction in atrial size (control 10,960 ± 320 µm^2^, n = 14; *nrp2b* MO 5,314 ± 670 µm^2^, n = 10; Mann Whitney, p < 0.0001), suggesting an early role for Nrp2b that precluded its analysis in later heart development. Thus, we only assessed *plxna3*-deficient embryos. Importantly, similar to the *sema3fb* mutants, *plxna3* morphants exhibited smaller-sized atria than control, as evidenced by *myh6* (N = 1, control n = 5/5 normal; *plxna3* MO n = 9/15 small, Fig. 7A, B) and *tnnt2a* (N = 1, control n = 7/8 normal; *plxna3* MO n = 7/10 small, data not shown) expression. In support, *plxna3* MO atria were significantly smaller than those in control (N = 2; control 11,749 ± 420 µm^2^, n = 13; *plxna3* MO 9,142 ± 355 µm^2^, n = 17; Mann Whitney, *p* =  < 0.0001, Fig. 7F). Similar to *sema3fb*^*ca305*^ embryos, *plxna3*-deficient 72 hpf embryos showed a reduction in heart rate (99.2 ± 1.9 beats/min, N = 3, n = 10; Mann Whitney, *p* = 0.011, Fig. 7G) as compared to controls (111.1 ± 3.1 beats/min, N = 3, n = 9). Cardiac output (control 20.8 ± 2.9 nL/min, N = 3, n = 9; *plxna3* MO 14.6 ± 1.8 nL/min, N = 3, n = 10; Mann Whitney, *p* = 0.09; Fig. 7H) and ejection fraction (control 42.9 ± 4.1%, N = 3, n = 9; *plxna3* MO 34.4 ± 2.2%, N = 3, n = 10; Mann Whitney, *p* = 0.18; Fig. 7I) were not different between morphant and control hearts, despite the presence of edema in the *plxna3* morphants (data not shown). Likely the partial loss-of-function produced with the *plxna3* MO caused less severe cardiac defects than those seen in the *sema3fb* mutant, but that over time resulted in edema.

It was important to determine whether in addition to the smaller heart, the key alterations observed in chamber development present in *sema3fb* mutants were also present to some degree in the *plxna3* morphants. Indeed, the area (control 76.7 ± 4.1 µm [2], N = 2, n = 6 hearts; *plxna3* MO 54.6 ± 5.2 µm [2], N = 2, n = 11; Mann Whitney, *p* = 0.019; Fig. 7J) and perimeter (control 37.8 ± 1.1 µm, N = 2, n = 6; *plxna3* MO 31.9 ± 1.3 µm, N = 2, n = 11; Mann Whitney, *p* = 0.014; Fig. 7K) of DM-GRASP + ventricular cardiomyocytes were smaller in morphants than controls. Circularity of the morphant cardiomyocytes was comparable to controls (control 0.68 ± 0.02, N = 2, n = 6; *plxna3* MO 0.72 ± 0.02, N = 2, n = 10; Mann Whitney, *p* = 0.15; Fig. 7L). Notably, the distinct border of gene expression between the ventricle and atrium was also less evident in the *plxna3* morphants, as seen by the presence of strong *tnnt2a* expression in the atria (Fig. 7E) and ectopic *bmp4a* expression in the ventricle (Fig. 7C, D), as quantitated by measuring the area of the *bmp4a* AVC domain (WT 2,162 ± 210 µm^2^, n = 12; 250 µM *plxna3* MO 3,456 ± 472 µm^2^, n = 7; *p* = 0.028, Mann–Whitney). Together, these data argue that Plxna3 functions downstream of Sema3fb in the developing cardiac system. The *plxna3* morphant heart is impacted less severely than that of the *sema3fb* mutant, presumably because of residual Plxna3 signaling in the morphants.

## Discussion

Our data argue that embryonic cardiomyocytes secrete Sema3fb that spatially restricts chamber-specific gene expression at the atrioventricular border to ensure proper function of the embryonic heart. We find *sema3fb* mRNA is expressed by all cardiomyocytes over the period of chamber morphogenesis and differentiation, while expression of mRNAs for the candidate receptors, Nrp2b and Plxna3, is restricted predominantly to ventricular cardiomyocytes by 48 hpf. We propose that Sema3fb informs ventricular cardiomyocytes of their location at the border with atrial cells, and that the neighboring atrial cells likely lack the machinery to sense Sema3fb. The consequence is the proper refinement of chamber-specific gene expression at the AVC, a feature that is disrupted in the absence of Sema3fb. The size of both heart chambers is smaller, we propose because of these defects in gene expression. Our work argues for the first time that a cardiomyocyte-derived secreted Sema3 signals potentially in a spatially-restricted manner to promote the normal refinement of the border between the ventricle and atrium, a process required for proper chamber size and function.

We suggest a model whereby cardiomyocyte-derived Sema3fb signals to Plxna3/Nrp2b-expressing ventricular cardiomyocytes. In support, *sema3fb* mRNA is expressed by early cardiomyocytes in both heart chambers, while at 48 hpf mRNAs for Plxna3 and Nrp2 appear restricted largely to the ventricular cardiomyocytes. Of note, these receptor mRNAs also appear restricted to a subregion of the 28 hpf heart tube, though we did not directly assess their expression by ventricular vs. atrial cardiomyocytes at this stage. Further, the timing of heart phenotype presentation in the *sema3fb* mutants argues against the causative involvement of non-cardiomyocyte tissues. While Purkinje fibers secrete SEMA3A that patterns sympathetic innervation of the murine pacemaker node [20], Sema3fb in zebrafish likely does not play a similar role, at least at the 48 hpf analysis time point, in that the cardiomyocyte phenotypes arise prior to when the node is innervated [76]. Epicardial cells also arrive in the heart after clear chamber defects are evident in the mutants at 48 hpf: epicardial cells emerge at around 55 hpf and migrate to and attach to the myocardium by 72 hpf [39]. We also saw in mutant hearts the normal arrival of cardiomyocytes from the secondary heart field [56] and CNC [77]. Interestingly, Sema3fb is important in zebrafish epicardial development at later stages: by 5 dpf *sema3fb* is expressed by *tbx18* + epicardial cells and not cardiomyocytes, and Sema3fb regulates epicardial cell numbers in the bulbus arteriosus [22]. Thus, Sema3fb has at least one additional role in heart development than the early function we describe here.

Endocardial cells are important in cardiomyocyte development. For instance, PLXND1 signaling within the murine endocardium promotes cardiomyocytes to form trabeculae [78]. Yet, our data argue that alterations in cardiomyocytes in *sema3fb* mutants do not occur secondary to defects in the adjacent endocardial cells. First, the *notch1b* and *nfatc1* AVC endocardial markers are expressed at 48 hpf in *sema3fb* mutants. Second, in both WT and *sema3fb* mutants the *vegfr2/kdrl*-expressing endocardium lines both heart chambers, and the endocardium-derived heart valves form. The mutant valves do appear mis-distributed at the atrioventricular border, possibly as a result of altered gene expression at the atrioventricular border, but this is a mild phenotype. Third, the cardiomyocytes and not the endocardium express mRNAs for the known Sema3f receptors, Plxna3 and Nrp2 [79]. Nor do we think Sema3fb acts on endothelial cells via Vegfr2, as SEMA3F inhibition of VEGF signaling occurs via NRP and not VEGFR [80, 81], and *nrp* mRNA appears to be absent from the early zebrafish endocardium [50]. Finally, we saw no sustained defects in heart tube assembly, a process known to be influenced by the endocardium [82]. These data argue that the contribution of the endocardium to the initial heart impairments we observe in *sema3fb* mutants is minimal. Instead, the literature and our data strongly support the idea that cardiomyocyte defects arise from the loss of Sema3fb signaling within the cardiomyocytes themselves. Nonetheless, it is possible that defects in valve morphogenesis, and subsequent anatomy and function contribute to the cardiac dysmorphogenesis and edema seen in the *sema3fb* mutants.

Our data argue against a Sema3fb signal promoting directly a ventricular fate or repressing atrial/AVC fates. While both *sema3fb* and receptor mRNAs are expressed within the early bilateral heart field, ventricular and atrial cardiomyocyte progenitors are specified much earlier, prior to zebrafish gastrulation [83]. Indeed, both the formation of the ventricular and atrial chambers and their subsequent morphogenesis appear to occur normally in the absence of Sema3fb. Furthermore, *irx1a* and *tbx5a*, transcription factors implicated in ventricular cardiomyocyte differentiation, remain expressed throughout the mutant ventricle. Thus, if Sema3fb has an early role in heart development, it likely acts redundantly with other mechanisms.

The main impact of Sema3fb loss occurs after the initial specification of the chambers. Interestingly, Tolterodine-mediated blockade of the cardiac conduction system from the 10 ss to 48 hpf results in a small ventricle in the absence of alterations in cell death and ventricular cardiomyocyte numbers [84], similar to what we see in *sema3fb* mutants. Yet, other aspects of the mutant and Tolterodine-treated hearts differ, including the complete loss of *notch1b* mRNA at the AVC and a normal sized atrium in the latter, but not the former. Thus, the *sema3fb* mutant phenotype is not explained simply by the loss of the cardiac conduction system. Both atrial and ventricular chambers are smaller in the absence of Sema3fb, and several genes and proteins whose expression normally obeys the border between the atrium and ventricle are disrupted: 1) *bmp4a* and *tbx2b*, which are expressed by the specialized slow-conducting cardiomyocytes of the AVC [85] by 48 hpf, fail to show this restriction in mutants (*bmp4a* label at 72 hpf argues that ultimately this refinement occurs), 2) *myh7* is largely absent from the ventricle immediately proximal to the border, 3) *myh6* spreads anteriorly past the atrioventricular morphological constriction, and 4) DM-GRASP, MF20, and *tnnt2a* mRNA that are normally expressed more robustly in the ventricle show no dramatic change in expression at the border between the ventricle and atrium. These data support the idea that Sema3fb provides a patterned signal to ventricular cardiomyocytes at the border with the atrial chamber. This signal does not establish the border between the chambers, as the AVC does form in the *sema3fb*^*ca305*^ heart. Further, several genes still exhibit the expected profound change in expression at the border, including *irx1a*, *bmp4a* and *myh6*, though the expression border at the AVC for all three genes is imprecise and jagged.

Instead, we propose that Sema3fb allows for the sharpness of the border to emerge in a timely manner. Ventricular cardiomyocytes in the border region can respond to Sema3fb while immediately adjacent atrial cardiomyocytes lack the receptors to do so. Our data suggest that this differential signaling ability is required for the refinement of the AVC region. The overexpression data support the idea that Sema3f tells ventricular cardiomyocytes that they sit at the border with the atrium. Certainly, the fact that ectopic *bmp4a* expression in the 48 hpf heart is seen with both loss- and gain- of function of Sema3f argues against the possibility that Sema3fb regulates *bmp4a* transcription. Instead, our data suggest that if the correct spatial information provided by Sema3fb is lost, either because the signal itself is gone (mutant) or the signal is disrupted by cells in the AVC region experiencing different levels of signaling (overexpression), proper refinement of the AVC region fails to occur. An imprecise AVC region leads to disrupted regulation of cardiomyocyte-specific genes. In support, restriction of Bmp signaling to the AVC is necessary to prevent inhibition of ventricular cardiomyocyte differentiation by Tbx2 [86–88].

Interestingly, in *sema3fb* mutants the atrium is also impacted in size and misexpression of ventricular and AVC markers. Given that many atrial cardiomyocytes may lack the machinery to respond to Sema3fb, altered atrial differentiation arises presumably secondarily to disrupted ventricular cardiomyocyte and/or AVC development. One possibility is that disrupted ventricle function impacts atrial cardiomyocytes, as the function of the two chambers shows some inter-dependence [59]. Explanted mutant atria are still smaller than their WT counterparts, however, despite having removed flow as a variable. Potentially, alterations in the secretion of factors from ventricular cardiomyocytes and/or cells of the AVC affects the differentiation of the nearby atrial cardiomyocytes. Alternatively, the presence of *plxna3* mRNA at 48 hpf in the anterior pole of the atrium may indicate a potential direct role for Sema3f signaling in atrial development.

The heart chambers are smaller in *sema3fb* mutants, though the data suggests that fewer cardiomyocytes is not the explanation. The expression of *fgf8a*, *tbx5a* and *nkx2*.5 in the primary heart field, and markers of secondary heart field-derived cells, are comparable in the two genotypes. Further, cell death and proliferation at 48 hpf are not impacted by Sema3fb loss. Indeed, similar numbers of ventricular cardiomyocytes are present in WT and mutant hearts at this stage. Instead, without Sema3fb these cardiomyocytes appear to be smaller than normal. The fact that the atrial chamber was smaller in the absence of changes in proliferation, apoptosis and cell integration also argues for smaller cardiomyocytes within the atria. Heart dysfunction could result in smaller cells, in that zebrafish cardiomyocytes hypertrophy normally at about 45 hpf in response to shear stresses arising from the onset of blood flow through the heart [72]. Yet, the atria of explanted mutant hearts were still smaller than those of WT, even though shear force was removed as a functional regulator. Potentially, Sema3fb signaling causes cytoskeletal rearrangements that induce ventricular cardiomyocyte expansion. For atrial cardiomyocytes, and possibly their ventricular counterparts, disrupted expression of select differentiation genes remains a potential explanation for the failure of cardiomyocytes to undergo hypertrophy.

While a significant portion of the hearts of *sema3fb* mutants are edematous at 48 and 72 hpf, the vast majority of embryos (> 90%) develop to adulthood and are fertile. Potentially the ability to recover from the loss of early Sema3fb relates to the fact that parameters of heart function (e.g. ejection fraction) altered in the *sema3fb* mutants associate with the size of the heart chambers, and not the function of the heart as a pump. Chamber size may recover with time in larval fish, either via cardiomyocyte hypertrophy or the addition of new cardiomyocytes. An interesting possibility to address in the future is whether *sema3fb* mutant fish are nonetheless more sensitive to cardiac stress, as has been seen with knockdown of *Vezf* in zebrafish. [89].

*Sema3f* is expressed in the cardiac cushions and in scattered cells throughout the myocardium of the embryonic chick heart [90], and scRNAseq data suggests that *Sema3f*, *Nrp2* and *Plxna2* mRNAs are expressed in developing mouse cardiomyocytes [91]. Targeted disruption of mouse Sema3f results in partially penetrant embryonic lethality likely as a result of defective vascularization of the placenta [92]. The placental defect complicates analysis of heart phenotypes in null animals. An embryonic role for SEMA3F in the human heart has not been described. A polymorphism in *SEMA3F* is associated with myocardial infarction [93]. While this association may not relate to a role for SEMAF in human fetal heart, congenital heart defects have been linked to defects in SEMA signaling [94, 95].

## Conclusions

Here we provide evidence for a secreted Sema functioning as an extrinsic regulator of regional differentiation in the cardiovascular system. Sema3s are known to be important for development of the mouse cardiovascular system [16]. In some cases, the roles are myocardium-independent, as is the case for SEMA3A and innervation of the heart [20], and Sema3d and SEMA3C in CNC-dependent generation of cardiomyocytes [96, 97]. Here, we suggest a novel cardiomyocyte autonomous function of Sema3fb in governing the molecular differentiation of cardiomyocytes. In mouse, transmembrane SEMA6D promotes cardiomyocyte proliferation [98]. In concert with our findings, these data support the idea that Semas directly regulate cardiomyocyte development. Additional roles for Semas will likely be identified in heart development as more subtle cardiovascular defects are assessed in loss-of-function models.

## Supplementary information

**Additional file 1.** Supplementary Data.

## Data Availability

The datasets used and/or analysed during the current study are available from the corresponding author on reasonable request.

## Abbreviations

Sema: Semaphorin
RT-PCR: Reverse transcriptase polymerase chain reaction
Nrp: Neuropilin
Plxn: Plexin
Bmp: Bone morphogenetic protein
Fgf: Fibroblast growth factor
hpf: Hours post-fertilization
WT: Wild type
MS-222: Tricaine methanesulfonate
ISH: In situ hybridization
PFA: Paraformaldehyde
PBS: Phosphate buffered saline
DSHB: Developmental Studies Hybridoma Bank
H&E: Hematoxylin and Eosin
SEM: Standard error of the mean
SD: Standard deviation
AVC: Atrioventricular canal
Suppl Fig: Supplemental Figure
*tnnt2a*: *cardiac troponin T type 2a*
sgRNA: Single guide RNA
bp: Base pair
aa: Amino acid
MO: Morpholino oligonucleotide
myh: Myosin heavy chain
Irx: Iroquois
Tbx: T-box transcription factor
CNC: Cardiac neural crest
Crip: Cysteine-rich intestinal protein
Ltbp: Latent tgfB binding protein
