# Supplementary material for: Semaphorin3f as a cardiomyocyte derived regulator of heart chamber development
Source: Cell Commun Signal. 2022 Aug 19;20:126. doi: 10.1186/s12964-022-00874-8 (PMC9389736; doi:10.1186/s12964-022-00874-8)
Supplement: Supplementary file 2 — Additional file 1. Supplementary Data. [file 12964_2022_874_MOESM2_ESM.docx]

**
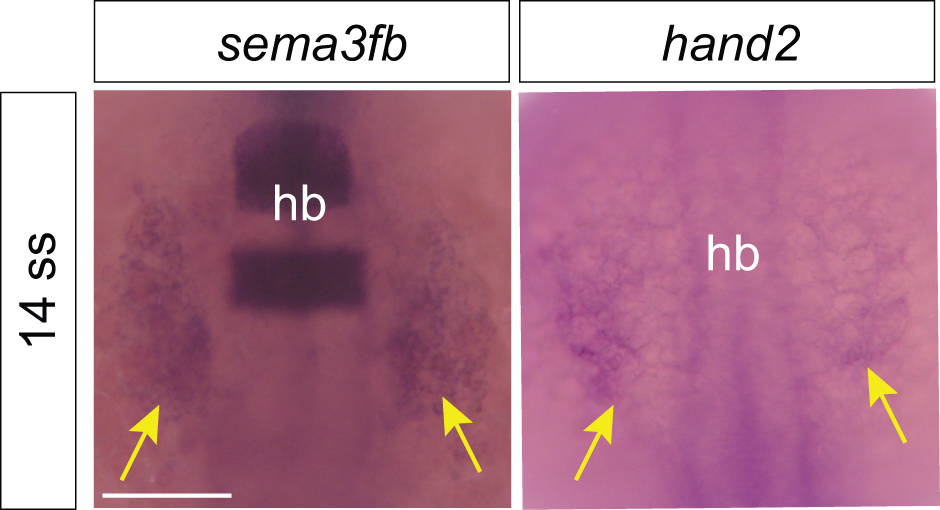
**

**Supplementary Figure 1: *sema3fb* mRNA is expressed in the early heart field.** Dorsal view of whole mount ISH for *sema3fb* mRNA at the 14 somite stage in the early heart field (yellow arrows) as marked by *hand2*. hb, hindbrain. Scale bar is 50 µm.

**
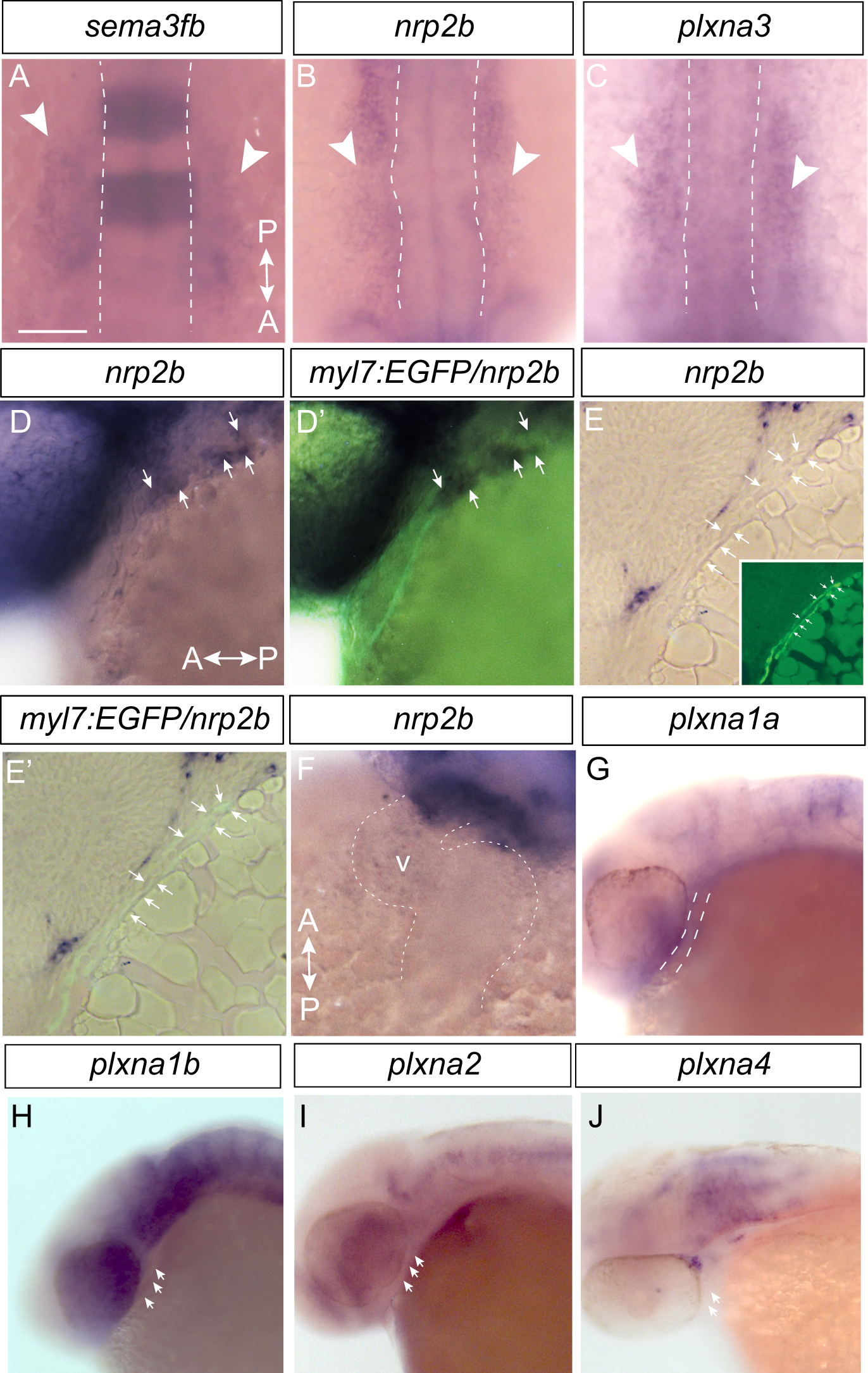
**

**Supplemental Figure 2: Expression of *nrps* and *plxns* in early heart development. A-C)** Dorsal views of ISH for *sema3fb*, *nrp2b* and *plxna3* at 18 hpf, revealing presumptive expression in the heart field (white arrowheads). **D-F)** *nrp2b* whole mount ISH signal in the 28 hpf (D,E) and 48 hpf (F) heart. *nrp2b* mRNA (D,E) and the blend of *nrp2b* ISH signal with EGFP (D’,E’) in a *Tg(myl7:EGFP)* heart, indicating expression in cardiomyocytes (arrows). Inset in E shows GFP expression at low power. Expression of *nrp2b* mRNA in the ventricle at 48 hpf (F). **G-J)** *plxna* ISH shows minimal signal in the 28 hpf heart (white arrowheads). A, anterior; P, posterior; v, ventricle. Orientation in A is for A-C, in D is for D-E and G-J. Scale bar in A is 50 µm (A-F), 100 µm (G-J).

**
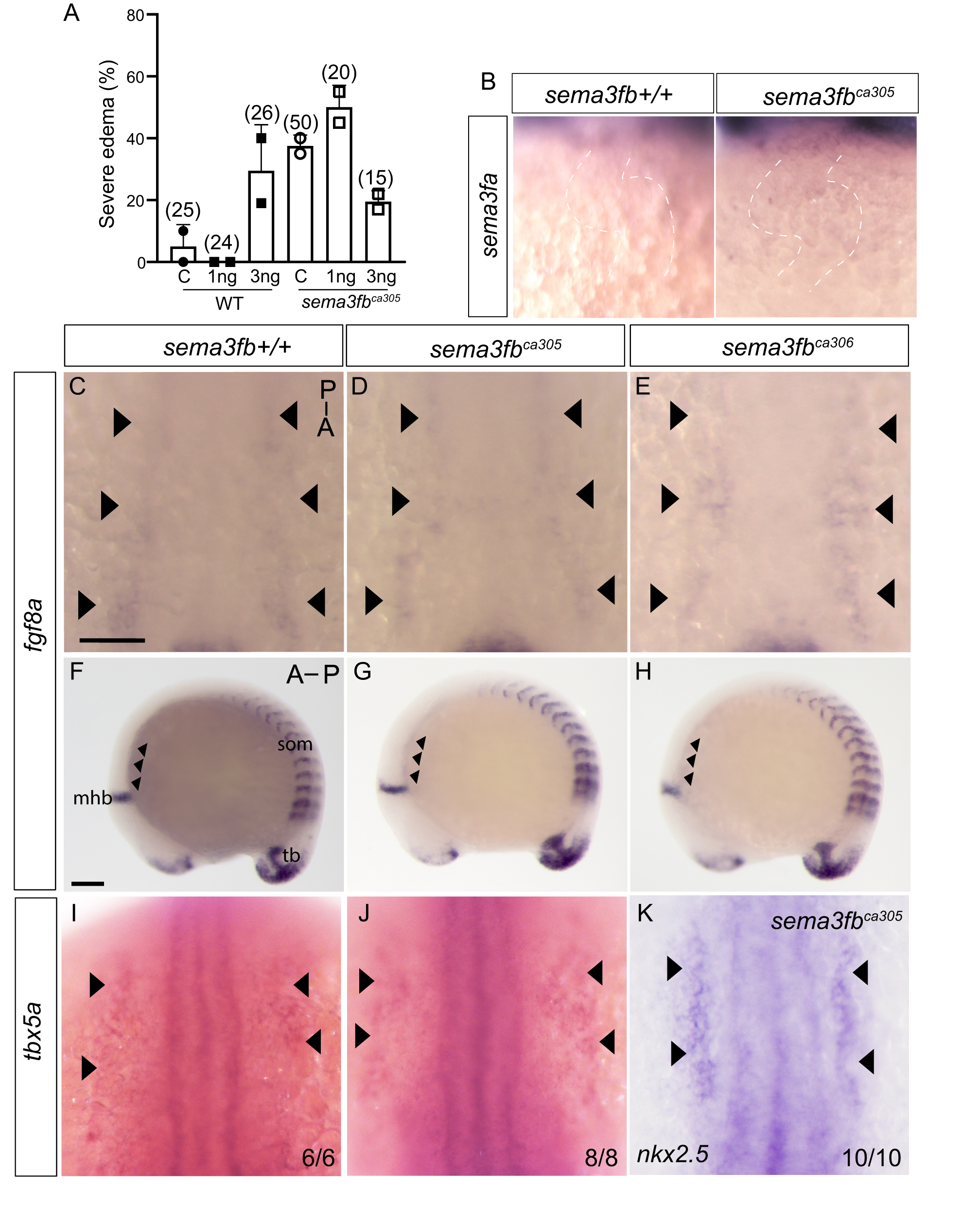
Supplementary Figure 3: Early heart development occurs normally with Sema3fb loss. A)** Percentage of embryos with a severe heart edema in WT and *sema3fb^ca305^* 48 hpf embryos and those injected at the one-cell stage with either water control (C) or 1-3 ng of a *sema3fb* ATG antisense morpholino oligonucleotide. (N=2; numbers of embryos in brackets). **B)** Little or no *sema3fa* mRNA in the 48 hpf WT (n=4) or *sema3fb^ca305^* heart (n=5). **C-H)** Dorsal (C-E) and lateral (F-H) views of 13 hpf WT (C,F), *sema3fb^ca305^* (D,G), and *sema3fb^306^* (E,H) embryos. *fgf8a* is expressed normally (N=2, n=10) in the heart field-containing anterior lateral plate mesoderm; arrowheads in low magnification lateral (F-H) and high magnification dorsal (C-E) views. **I-K)** Expression of early heart field markers *tbx5a* (I,J) and *nkx2*.5 (K). A, anterior; mhb, midbrain-hindbrain boundary; P, posterior; som, somites; tb, tailbud. Scale bar in C is 50 µm (C-E,I-K), and in F is 100 µm (F-H).

**
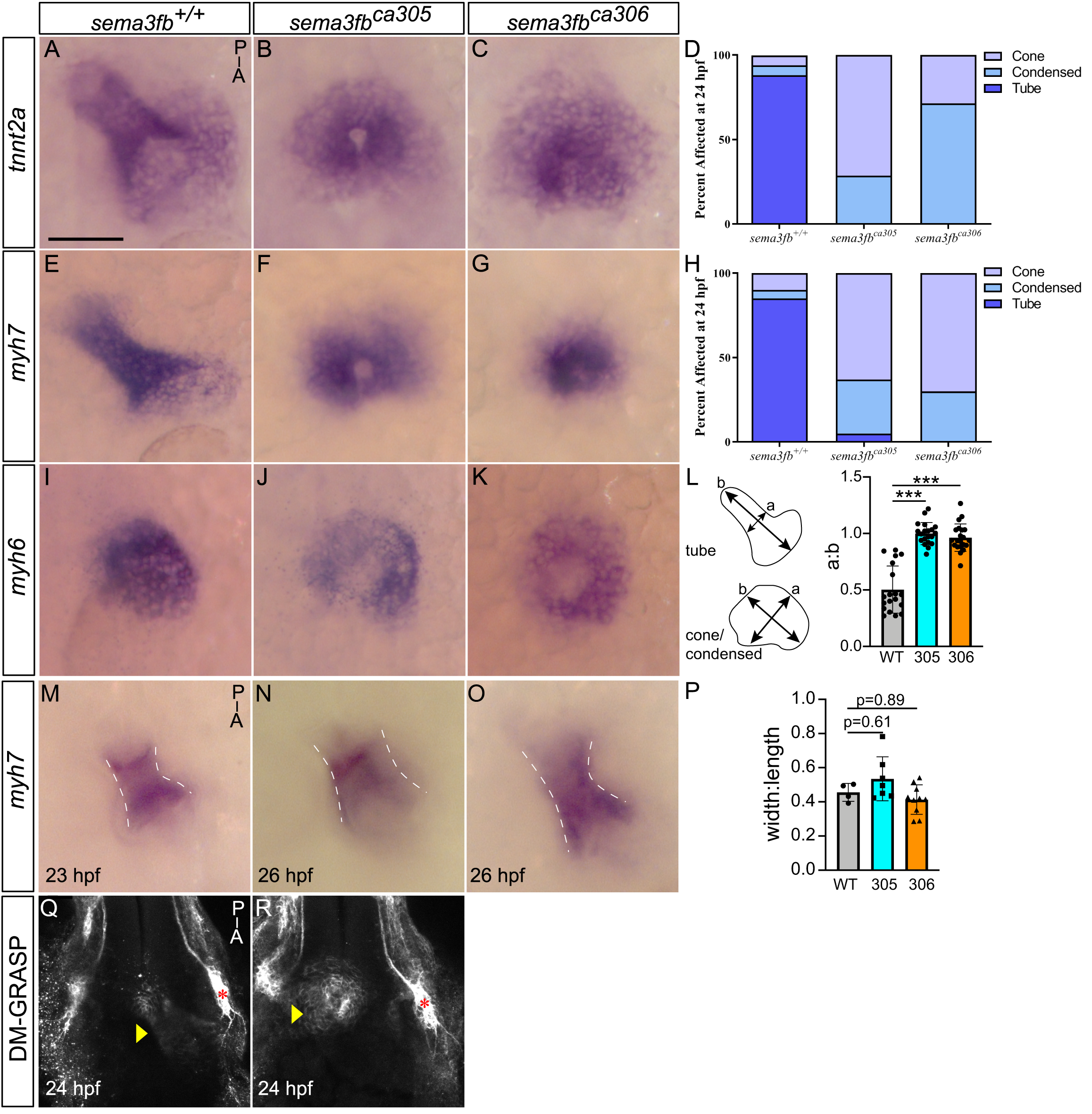
**

**Supplemental Figure 4. Heart tube extension is delayed slightly in *sema3fb^ca305^* embryos.** Ventral views of ISH processed WT, *sema3fb^ca305^* and *sema3fb^ca306^* 24 hpf embryos. **A-C)** *tnnt2a*. Linear heart tube in WT (N=3, n=15/17) (A), with cone (B) and condensed (C) morphologies in both mutant alleles (N=3, 14/21 *sema3fb^ca305^*; N=3, 15/21 *sema3fb^ca306^*). **D)** Percent occurrence of the different *tnnt2a* cardiac morphologies. **E-G)** Ventricular cardiomyocyte-specific *myh7*. Elongation of WT hearts (N=3, n=17/20) (E), while *sema3fb*-/- hearts exhibit cone or condensed morphologies (N=3, 12/19 *sema3fb^ca305^*; N=3, 13/19 *sema3fb^ca306^*) (F,G). **H)** Percent occurrence of the different *myh7* cardiac morphologies. **I-K)** Atrial cardiomyocyte-specific *myh6*. WT atria are tubularized (N=3, n=17/20) (I), but primarily at the cone stage in both mutant alleles (N=3, 19/20 *sema3fb^ca305^*; N=3, 11/18 *sema3fb^ca306^*) (J,K). **L)** Quantitation of the *tnnt2a* heart shape at 24 hpf in WT (N=2, n=19), *sema3fb^ca305^* (n=20) and *sema3fb^ca306^* (n=21) fish. Schematic shows the width (a) and length (b) measurements on a linear heart tube and a condensed heart. Graph represents the ratio of a:b. Kruskal-Wallis ANOVA, Dunn’s Multiple Comparisons test, p<0.0001. **M-O)** Ventricle specific marker *myh7* for WT at 23 hpf (N=1, n=4/4, M), and at 26 hpf for *sema3fb^ca305^* (N=1, n=7/7, N) and *sema3fb^ca306^* (N=1, n=10, O). **P)** Quantitation of the *myh7*+ ventricle shape (ratio of width to antero-posterior length) at 23 hpf in WT (n=4), and 26 hpf for *sema3fb^ca305^* (n=7) and *sema3fb^ca306^* (n=10) fish. Kruskal-Wallis ANOVA, Dunn’s Multiple Comparisons test, p=0.0805. **Q-R)** DM-GRASP immunostaining shows cranial ganglion (red asterisk) that has migrated and formed normally in WT (n=4, Q) and *sema3fb^ca305^* (n=4, R) 24 hpf embryos, despite the heart of mutants showing a mild developmental delay (yellow arrowhead). A: anterior; P: posterior. Scale bar: 100 μm.

**
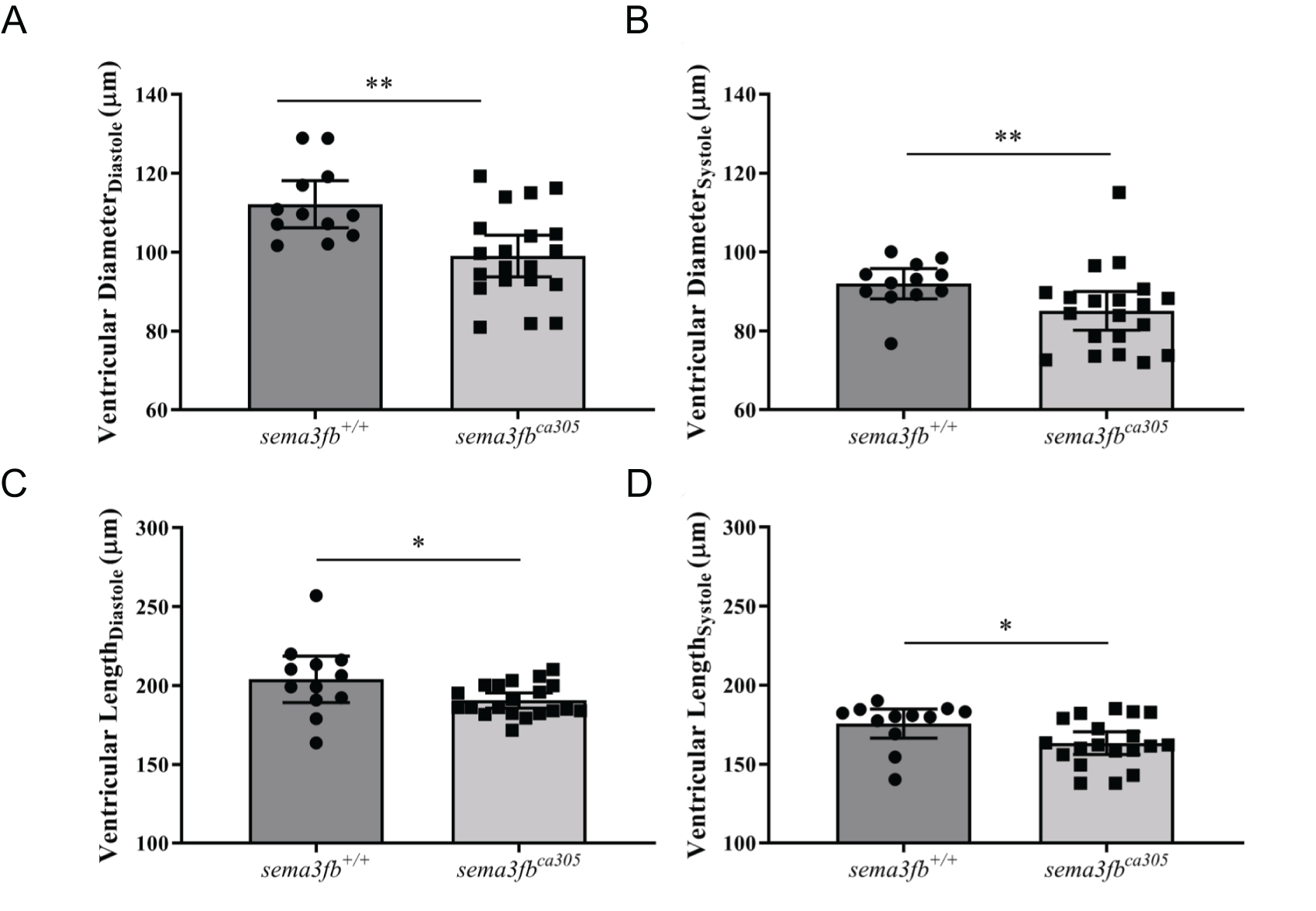
Supplemental Figure 5. Ventricle size is reduced significantly in *sema3fb* mutants.** **A-D)** Ventricle measurements made from still images taken during video microscopy of WT and mutant 72 hpf hearts. Quantitation reveals that in mutants as compared to WT the ventricle diameter during diastole (A, p=0.0017) and during systole (B, p=0.0063) is significantly smaller, and ventricle length is significantly shorter at diastole (C, p=0.048) and systole (D, p=0.031). N=3; n=12 WT, n=20 *sema3fb^ca305^*.

**
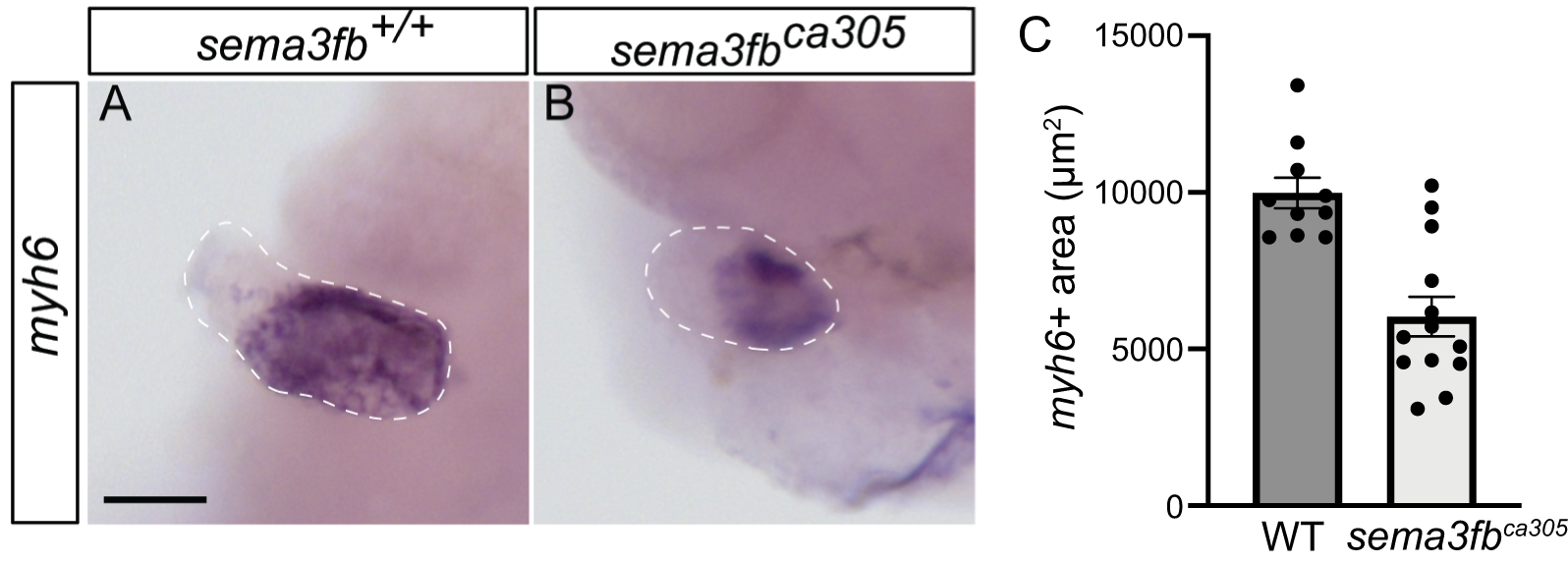
Supplemental Figure 6. Reduced cardiac chamber size in *sema3fb* mutant occurs in the absence of blood flow.** Heart explants dissected from embryos at 24 hpf and cultured for 24 hours before processing for *myh6* ISH (**A,B**). The *sema3fb^ca305^* atria (N=2, n=10/13) are smaller in size than WT (N=2, n=10/10). **C)** Graph of atrial area (p=0.0011, Mann Whitney). Scale bar: 50 μm.
